# Reduction in the levels of CoQ biosynthetic proteins is related to an increase in lifespan without evidence of hepatic mitohormesis

**DOI:** 10.1038/s41598-018-32190-y

**Published:** 2018-09-18

**Authors:** María Rodríguez-Hidalgo, Marta Luna-Sánchez, Agustín Hidalgo-Gutiérrez, Eliana Barriocanal-Casado, Cristina Mascaraque, Darío Acuña-Castroviejo, Margarita Rivera, Germaine Escames, Luis C. López

**Affiliations:** 1 Institute of Biotechnology, Biomedical Research Centre and Department of Physiology, Faculty of Medicine, University of Granada, Granada, Spain; 2 Centro de Investigación Biomédica en Red de Fragilidad y Envejecimiento Saludable (CIBERFES), Granada, Spain; 3 Institute of Neurosciences, Biomedical Research Centre and Biochemistry and Molecular Biology II, University of Granada, Granada, Spain

## Abstract

Mitohormesis is an adaptive response induced by a mild mitochondrial stress that promotes longevity and metabolic health in different organisms. This mechanism has been proposed as the cause of the increase in the survival in *Coq7*^+/−^ (*Mclk1*^+/−^) mice, which show hepatic reduction of COQ7, early mitochondrial dysfunction and increased oxidative stress. Our study shows that the lack of COQ9 in *Coq9*^*Q95X*^ mice triggers the reduction of COQ7, COQ6 and COQ5, which results in an increase in life expectancy. However, our results reveal that the hepatic CoQ levels are not decreased and, therefore, neither mitochondrial dysfunction or increased oxidative stress are observed in liver of *Coq9*^*Q95X*^ mice. These data point out the tissue specific differences in CoQ biosynthesis. Moreover, our results suggest that the effect of reduced levels of COQ7 on the increased survival in *Coq9*^*Q95X*^ mice may be due to mitochondrial mechanisms in non-liver tissues or to other unknown mechanisms.

## Introduction

Aging, at a biological level, is associated with the gradual accumulation of a wide variety of molecular and cellular damage. Over time, this damage leads to a gradual deterioration in physiological functions and an increased risk of aging-associated diseases, resulting in death^1^.

The study of the underlying mechanisms of aging has attracted scientific interest and multiple theories have been proposed to explain this phenomenon. Among those theories, two of them interconnect the generation of reactive oxygen species (ROS) and mitochondrial damage with the cellular senescence. In the 1950s^2^, Denham Harman proposed the free radical theory of aging, suggesting that free radicals induce accumulation of damage in the cell over time making it the cause of aging and a major determinant of lifespan^3^. In the 1970s, Harman published an extension of this theory, the mitochondrial theory^4^, which specifies that mitochondria are both the primary source and the target of ROS. Mitochondria produce ROS during oxidative phosphorylation and those ROS cause oxidative damage to mitochondrial macromolecules such as mtDNA, proteins or lipids, inducing aging^3,5,6^. Also mitochondria participate in the apoptosis regulation and age-related mitochondrial oxidative stress may contribute to apoptosis^5^.

Several studies have supported such theories, but there are other studies with different conclusions as well. Additionally, the connection between ROS and aging has suffered some changes in the last decades because some data from model organisms suggest that early oxidative stress may promote longevity and metabolic health through the concept of mitochondrial hormesis or mitohormesis^3,5,7^. In general, hormesis is defined as any adaptive response exhibiting a biphasic dose response characterized by a beneficial effect in low doses and an inhibitory or toxic effect in high doses. Therefore, mitohormesis is a particular type of hormesis in which a mild mitochondrial stress can induce an adaptive response of cells and organisms that potentially determine how long they live^8,9^.

In many organisms, like yeast, worm, flies and mice, it has been observed that a mild inhibition of mitochondrial respiration in the early stage of life extends their lifespan^10^. This has been achieved under condition of caloric restriction, exercise or hypoxia, as well as in specific mutations in some component related to the mitochondrial respiratory chain^11^. For example, mutational inactivation of *clk-1* in *Caenorhabditis elegans* and or partial inactivation of its orthologue *Mclk1* in mouse liver increases longevity in these organisms^11–13^.

CLK-1/MCLK1 (=COQ7) is a mitochondrial hydroxylase involved in Coenzyme Q (CoQ) biosynthesis^13^. CoQ is a mobile electron carrier in the mitochondrial respiratory chain, a potent endogenous antioxidant and an essential lipid for the redox reaction of some mitochondrial proteins, i.e. Sulfide Quinone Oxidoreductase (SQOR) or Dihidroorotate Dehydrogenase. The synthesis of CoQ occurs mainly in mitochondria and involves at least eleven proteins (COQ1-COQ9, YAH1 and ARH1) that are organized in a multiprotein complex^14,15^. Recently, we have characterized a mouse model carrying a homozygous mutation in *Coq9* gene (Q95X, *Coq9*^*Q95X*^)^16^. This mutant has a premature termination in the COQ9 protein, which is needed for the stability and activity of COQ7, an enzyme that catalyzes the hydroxylation of demethoxyubiquinone (DMQ) to produce 5-hydroxyquinone (5-HQ)^17,18^. *Coq9*^*Q95X*^ mice have undetectable levels of COQ9 protein and, as a consequence, they have a moderate CoQ deficiency in the brain, kidneys and skeletal muscle. Also a proportion of COQ7 could be found in the nucleus, where it suppresses the mitochondrial unfolded protein response (UPR^mt^)^19^. However, this effect has not been tested in *Coq9* mutant mice.

Because the absence of COQ9 in the *Coq9*^*Q95X*^ mice induces a severe reduction in the levels of COQ7, a protein that has been related with the increase in life expectancy, we aim to measure the longevity on this mouse model and establish a connection with possible mitochondrial changes in the liver at different ages.

## Results

### Animal survival and body weight

**Figure 1:**
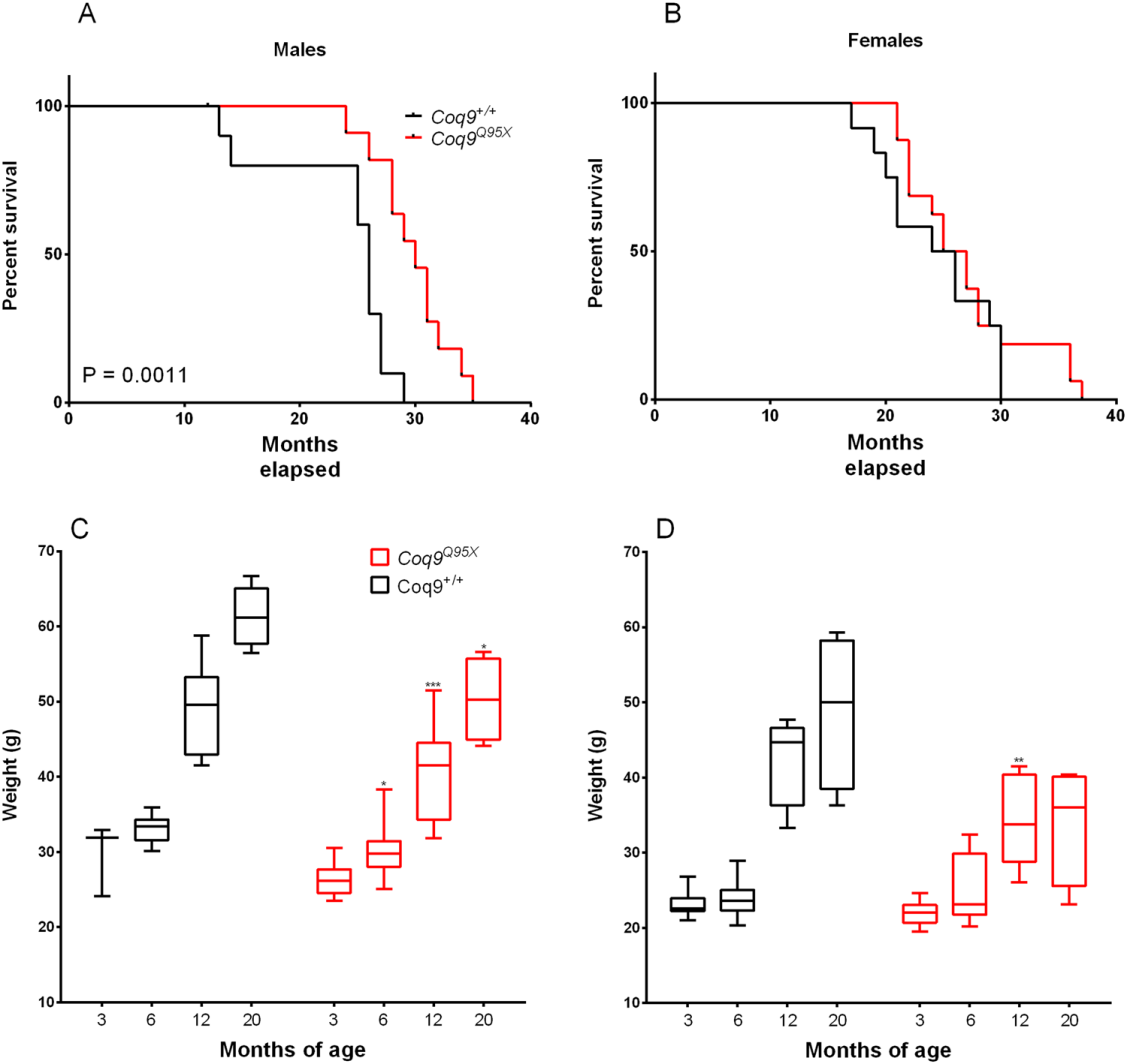
Animal Survival and body weight. (**A**,**B**) Kaplan-Meier survival curves of *Coq9*^*Q95X*^ and *Coq9*^+/+^ male and female mice, respectively, with *p* values calculated by the log-rank (Mantel-Cox) test and the Gehan-Breslow-Wilcoxon test. For the survival analysis, 23 *Coq9*^+*/*+^ mice (11 males and 12 females) and 26 *Coq9*^*R239X*^ mice (11 males and 16 females) were used. (**C**,**D**) Animal body weights of *Coq9*^*Q95X*^ and *Coq9*^+/+^ male and female mice, respectively. At each age, 5–13 animals were used in each sex. The graphs represent box & whisker plots that show a “box” with up edge at Q1, bottom edge at Q3, the “middle” of the box at Q2 (the median) and the maximum and minimum as “whiskers”.

Our group has previously demonstrated that *Coq9*^*Q95X*^ mice exhibit a severe reduction in the levels of COQ7 in the brain, kidneys and muscles^16^. In other animal models (nematodes and mice), the reduction in the levels of this protein has been related with an increase in life expectancy^12,13^. To test whether the reduction in COQ7 affects also the lifespan in *Coq9*^*Q95X*^ mice, we studied the survival in male and female of mutant and wild-type animals. We analyzed the survival by the log-rank (Mantel-Cox) test and the Gehan-Breslow-Wilcoxon test and found significantly greater survival of *Coq9*^*Q95X*^ males, *p* = 0.0011, and *p* = 0.0028, respectively (Fig. 1A). In males, the maximum lifespan was 35 months of age for the *Coq9*^*Q95X*^ mice (*n* = 11) and 29 months of age for the wild-type animals (*n* = 10); and *Coq9*^*Q95X*^ mice lived on average 15% longer than their wild-type littermates (30 vs. 26 months of age) (Fig. 1A). In females, there were no significant differences in survival, and *Coq9*^*Q95X*^ mice lived on average the same than their wild-type littermates (26 vs. 25 months of age) (Fig. 1B). Nevertheless, the maximum lifespan of female *Coq9*^*Q95X*^ mice was 37 months of age for the *Coq9*^*Q95X*^ mice (*n* = 12) and 30 months of age for the wild-type animals (*n* = 16).

The increase in lifespan in *Coq9*^*Q95X*^ mice came together with a progressive reduction in the animals’ body weight compared to wild-type animals (Fig. 1C,D). In females, this reduction is statistically significant at 12 months of age (Fig. 1D), while *Coq9*^*Q95X*^ males are thinner starting at 6 months of age (Fig. 1C).

### CoQ biosynthesis

**Figure 2:**
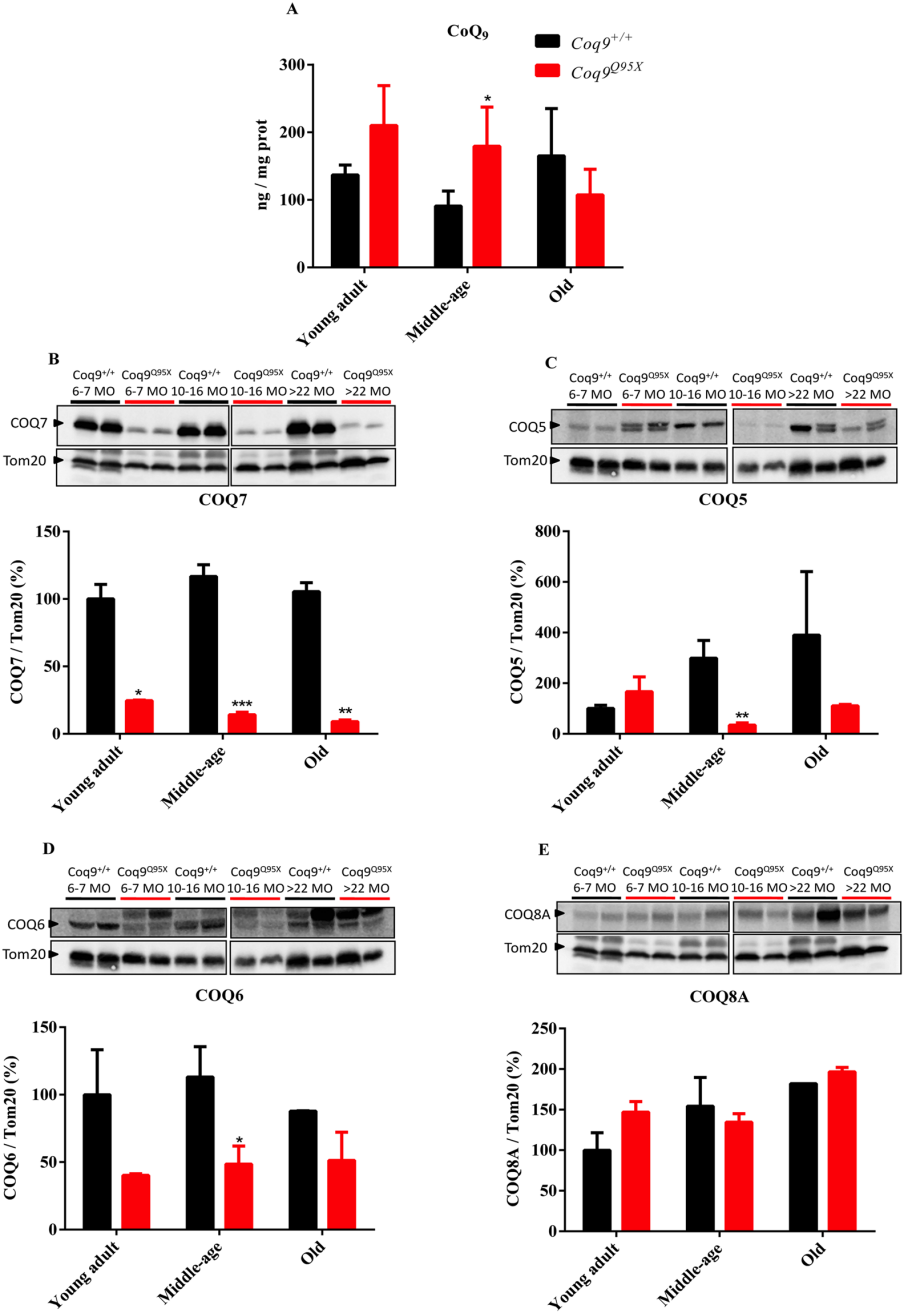
Levels of CoQ and the biosynthetic proteins COQ7, COQ5, COQ6 and COQ8A. (**A**) CoQ_9_ levels in liver homogenates of young adult, middle-age and old *Coq9*^*Q95X*^ and *Coq9*^+/+^ male mice. (**B**–**E**) Representative western blot of COQ7 (**B**), COQ5 (**C**), COQ6 (**D**) and COQ8A (**E**) and their quantitative analysis in the liver of young adult, middle-age and old *Coq9*^*Q95X*^ and *Coq9*^+/+^ male mice. *P < 0.05; **P < 0.01; *** *P* < 0.001; *Coq9*^*Q95X*^ versus *Coq9*^+/+^ littermates mice. The mitochondrial Tom20 was used as loading control. The specific band for each target protein is identified with an arrow, and only these bands were used in the quantification (the lower bands in COQ5 and COQ6). Note that the anti-COQ6 antibody recognizes two bands that may correspond to the full-length and mature (mitochondrial import) forms of COQ6 or, alternatively, to two different COQ6 isoforms^38,39^. Data information: (**A**–**E**) Data are expressed as mean ± SD. Statistical analyses were performed on *Coq9*^+/+^ male mice versus *Coq9*^*Q95X*^ male mice by Multiple Student’s *t-*test one per row. (**A**) *Coq9*^+/+^ mice, *n* = 6; *Coq9*^*Q95X*^ mice, *n* = 6, at each age (**B**–**E**) *Coq9*^+/+^ mice, *n* = 5; *Coq9*^*Q95X*^ mice, *n* = 5, at each age WB images from (**A–E**) were cropped from two different parts of the same gel at the same exposure.

Contrary to what occurs in other tissues (cerebrum, cerebellum, heart, kidney, extensor and *triceps surae*)^16^, we did not detect a reduction in the levels of CoQ_9_ in liver of *Coq9*^*Q95X*^ mice compared to wild-type mice. Even, in the middle-age group, the levels of CoQ_9_ were significantly higher in *Coq9*^*Q95X*^ mice than wild-type mice (Fig. 2A). In agreement with these data, the DMQ_9_ was not detected in our HPLC chromatographs in either *Coq9*^+*/*+^ or *Coq9*^*Q95X*^ (Fig. S1).

Because the levels of CoQ_9_ depend on the levels and activities of the CoQ biosynthetic proteins, we measured the levels of COQ7, COQ5, COQ6 and COQ8A in liver homogenates. In *Coq9*^*Q95X*^ mice, the levels of COQ7 were significantly decreased regardless of the age of the mice (Fig. 2B). However, the levels of COQ5 and COQ6 were only significantly reduced in the middle-age mutant mice (Fig. 2C and D), while COQ8A levels were unchanged (Fig. 2E).

### Mitochondrial bioenergetics

**Figure 3:**
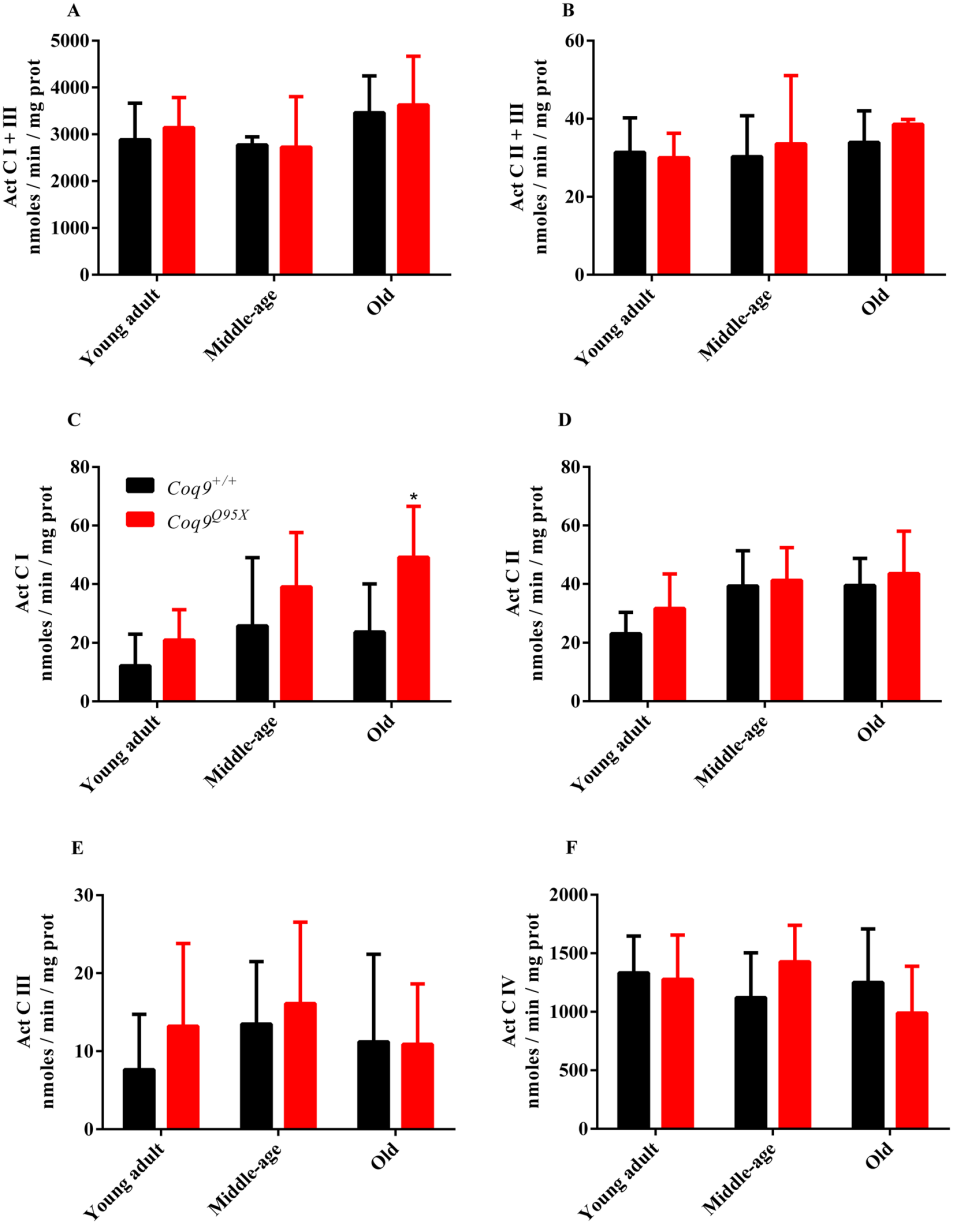
Mitochondrial respiratory chain activities. (**A**–**C**) CI + III (**A**), CII + III (**B**), CI (**C**), CII (**D**), CIII (**E**) and CIV (**F**) activities in liver homogenates of young adult, middle-age and old *Coq9*^*Q95X*^ and *Coq9*^+/+^ male mice. *P < 0.05; *Coq9*^*Q95X*^ versus *Coq9*^+/+^ littermates mice.Data information: (**A**–**F**) Data are expressed as mean ± SD. Statistical analyses were performed on *Coq9*^+/+^ male mice versus *Coq9*^*Q95X*^ male mice by Multiple Student’s *t-*test one per row. *Coq9*^+/+^ mice *n* = 6; *Coq9*^*Q95X*^ mice *n* = 6, at each age.

Our next step was to evaluate mitochondrial respiratory chain function in liver homogenates of *Coq9*^*Q95X*^ and control mice (Fig. 3). The activity of citrate synthase was also measured to correct the data by mitochondrial mass (Fig. 2S). Mitochondrial CI + III, CII + III, CI, CII, CIII and CIV activities were comparable in mutant and control mice (Figs 2S and 3), except for the higher CI activity observer in the mutant mice compared to wild-type mice.

Recent studies have shown that FGF21 is a hormone-like member of FGF family that responds to mitochondrial dysfunction and it is related with longevity and obesity^20^. Thus, we measured FGF21 protein level in *Coq9*^*Q95X*^ and *Coq9*^+/+^ male liver (young adult, middle-age and old). Two bands are detected by WB, the precursor and processed mature form of FGF21. There were no differences between precursor and mature forms inside each group of mice. Significant differences were only observed in the liver of old male mice, in which the mature form was diminished in the mutant mice (Fig. S3).

To test whether a mechanism involving the suppression of UPR^mt^ may participate in the survival increase, we measured the levels the mitochondrial proteins stress-70 protein (HSPA9 or GRP75) and ATP-dependent Clp protease proteolytic subunit (CLPP). Both proteins showed similar levels in *Coq9*^+/+^ and *Coq9*^*Q95X*^ mice at the three different ages (Fig. S4).

### Antioxidant defense and oxidative damage

**Figure 4:**
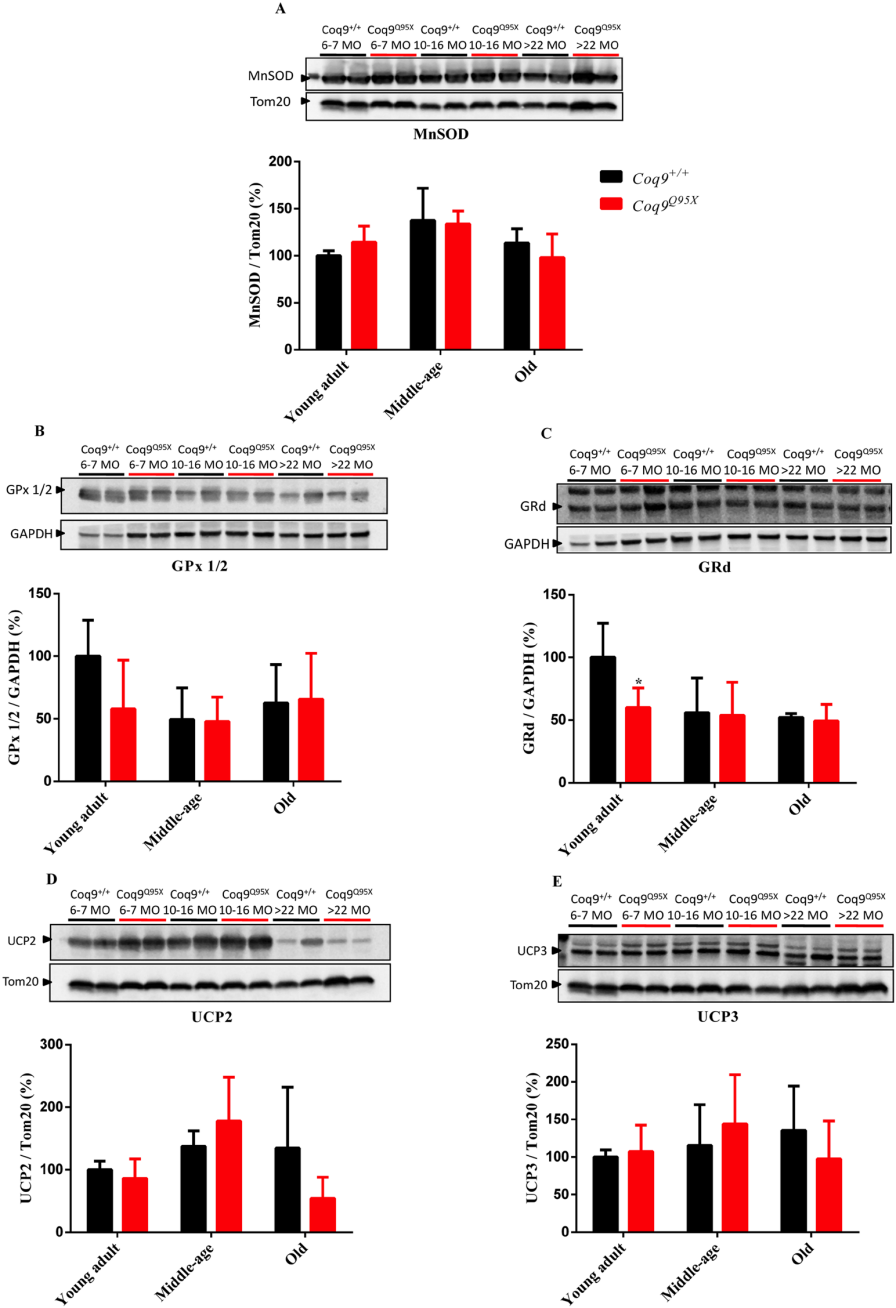
Proteins involved in antioxidant systems. (**A**) Representative western blot of MnSOD and their quantitative analysis (**A**) in the liver of young adult, middle-age and old *Coq9*^*Q95X*^ and *Coq9*^+/+^ male mice. *P < 0.05; **P < 0.01; ***P < 0.001; *Coq9*^*Q95X*^ versus *Coq9*^+/+^ littermates mice. The mitochondrial Tom20 was used as loading control. (**B**,**C**) Representative western blot of GPx1/2 (**B**) and GRd (**C**) and their quantitative analysis in the liver of young adult, middle-age and old *Coq9*^*Q95X*^ and *Coq9*^+/+^ male mice. *P < 0.05; **P < 0.01; ***P < 0.001; *Coq9*^*Q95X*^ versus *Coq9*^+/+^ littermates mice. The cytosolic GAPDH was used as loading control. The specific band for each target protein is identified with an arrow. (**D**,**E**) Representative western blot of UCP2 (**D**) and UCP3 (**E**) and their quantitative analysis in the liver of young adult, middle-age and old *Coq9*^*Q95X*^ and *Coq9*^+/+^ male mice. *P < 0.05; **P < 0.01; ***P < 0.001; *Coq9*^*Q95X*^ versus *Coq9*^+/+^ littermates mice. The mitochondrial Tom20 was used as loading control. The specific band for each target protein is identified with an arrow.Data information: (**A**–**E**) Data are expressed as mean ± SD. Statistical analyses were performed on *Coq9*^+/+^ male mice versus *Coq9*^*Q95X*^ male mice by Multiple Student’s *t-*test one per row. *Coq9*^+/+^ mice *n* = 6; *Coq9*^*Q95X*^ mice *n* = 6, at each age.

Because the reduction in COQ7 has been associated to changes in oxidative stress^13^, we first evaluated the levels of three antioxidant enzymes: MnSOD, GPx1/2 and GRd. The levels of MnSOD and GPx1/2 were similar in mutant and wild-type mice (Fig. 4A and B), and the only difference found was a reduction in GRd levels in young adult mutant mice compared to age-mated wild-type mice (Fig. 4C).

Then, we checked UCP2 and UCP3, which can prevent the generation of ROS by a mild uncoupling of the mitochondrial electron transport to the ATP synthesis^21^. The levels of both proteins were not statistically different in mutant and wild-type mice (Fig. 4D,E).

**Figure 5:**
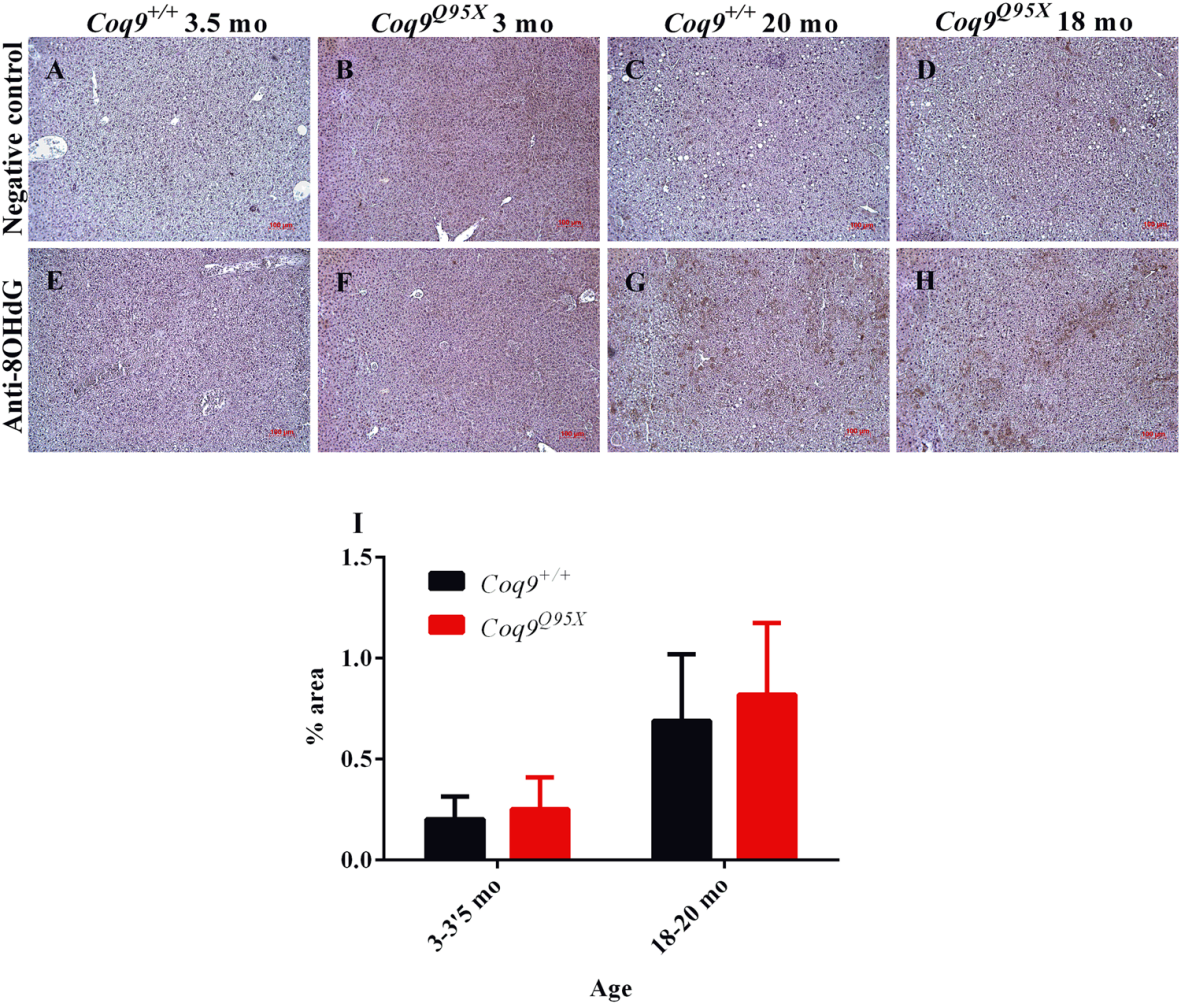
Inmunohistochemistry against 8-OHdG as a marker of oxidative damage. (**A**–**D**) Representative negative control of the liver at 3–3.5 month of age in *Coq9*^+/+^ (**A**) and *Coq9*^*Q95X*^ mice (**B**); and at 18–20 month of age in *Coq9*^+/+^ (**C**) and *Coq9*^*Q95X*^ (**D**) mice. (**E**–**H**) Representative anti-8OHdG immunohistochemistry of the liver at 3-3.5 month of age in *Coq9*^+/+^ (**E**) and *Coq9*^*Q95X*^ mice (**F**); and at 18–20 month of age in *Coq9*^+/+^ (**G**) and *Coq9*^*Q95X*^ (**H**) mice. Data information: Scale bars: 100 µm. (**I**) Percentage of 8OHdG positive signal in the images. Results correspond to the mean ± SD, as determined by the software ImageJ. *Coq9*^+/+^ mice *n* = 3; *Coq9*^*Q95X*^ mice *n* = 3, at each age.

We also tested the oxidation of DNA by immunohistochemical detection of 8-OHdG molecules. In correlation with the data of the antioxidant defense enzymes, we could not detect differences between mutant and wild-type mice, while an increased detection was observed during aging in both strains (Fig. 5).

## Discussion

Reduction in the levels of COQ7 in different organisms induces an increase in lifespan, a fact that has been attributed to the concept of mitohormesis^8,9^. Particularly, the increase in the life span in *Coq7*^+/−^(*Mclk1*^+/−^) mice has been associated to an early hepatic mitochondrial dysfunction, which induces a protective physiological response called mitohormesis^11^. Our study shows that *Coq9*^*Q95X*^ mice have a reduction in the levels of COQ7 and an increase in lifespan. However, the low levels of COQ7 does not induce a reduction in CoQ levels in the liver and, consequently, mitochondrial dysfunction is not observed in the liver of *Coq9*^*Q95X*^ mice.

*Coq9*^*Q95X*^ mice have a premature termination in the COQ9 protein that induces the activation of the nonsense-mediated mRNA decay (NMD) to avoid the expression of the aberrant *Coq9* mRNA^16^. As a consequence, *Coq9*^*Q95X*^ mice show undetectable levels of COQ9. Because COQ9 interacts with the hydroxylase COQ7, *Coq9*^*Q95X*^ mice show a severe reduction in the levels of COQ7 in the brain, kidneys, skeletal muscle and the liver. In the brain, kidneys and skeletal muscle, the reduction in COQ7 causes a decrease in the levels of CoQ^16^. However, in the liver, the low levels of COQ7 do not affect the levels of CoQ from 3 month to 22 month of age^16^. This phenomenon also occurs in *Coq7*^+/−^ mice^13^, and it can be explained by two possibilities: (1) the residual levels of COQ7 protein in the liver of *Coq9*^*Q95X*^ and *Coq7*^+/−^ mice are enough for the CoQ biosynthesis in liver, since the levels of CoQ in mouse liver are lower than the levels of CoQ in mouse kidney, heart, skeletal muscle and brain^16^; and (2) the existence of tissue-specific pathways or regulatory mechanisms for CoQ biosynthesis. Those options are supported by the fact that DMQ_9_ was not detected in the livers from *Coq9*^*Q95X*^ mice. Particularly, we can speculate that an alternative hepatic protein could also hydroxylases the DMQ. Alternative pathways in the biosynthesis of CoQ have been reported in yeasts in the last years^22–24^. Therefore, it seems that there are tissue-specific aspects in the CoQ biosynthetic pathway and its regulation, as it has been shown in the tissue-specific differences on the expression of CoQ biosynthetic genes in response to caloric restriction^25^.

The increase in longevity observed in the *Coq9*^*Q95X*^ mouse model is similar to the one reported in the *Coq7*^+/−^ mouse model. Nevertheless, the increase in the animal body weight over the months is reduced in *Coq9*^*Q95X*^ mice compared to wild-type mice, a fact that has not been reported in *Coq7*^+/−^ mice. Even, male *Coq7*^+/−^ mice are heavier than the male wild-type mice under 129Sv/J genetic background^11^. In concordance with our data, the administration of 2,4-dihydroxybenzoic acid (2,4-diHB) in wild-type mice reduces the animal body weight. 2,4-diHB is an structural analog of 4-dihydroxybenzoic acid (4-HB), the natural substrate for CoQ biosynthesis, that can partially inhibit the activity of COQ7 in normal cells with the subsequent reduction in CoQ levels and the increase of DMQ levels^26^. Therefore, the reduction of body weight in *Coq9*^*Q95X*^ mice and wild-type mice treated with 2,4-diHB could share similar mechanisms.

The proposed mechanism to explain the increase in lifespan in *Coq7*^+/−^ mice is the induction of a protective physiological event in response to a mild decrease in the function of the mitochondrial respiratory chain in the liver of young animals together with an increase in mitochondrial oxidative stress^13^. Because the levels of CoQ are normal in the liver of *Coq7*^+/−^ mice since 3 months of age, those changes in mitochondrial functionality and oxidative stress are not well understood. In the liver of *Coq9*^*Q95X*^ mice, however, the lack of changes in the mitochondrial bioenergetics, oxidative damage and antioxidant enzymes parameters correlates with the absence of CoQ deficiency^27,28^. Also, the levels of UCP2 and UCP3, two proteins that may be influenced by the levels of CoQ^29,30^, and that can protect from aging^29,30^, were similar in both *Coq9*^*Q95X*^ and wild-type mice. Therefore, the increase in lifespan in *Coq9*^*Q95X*^ cannot be attributed to a mitochondrial dysfunction in the liver of young animals unless this event occurs at a very early step in life. The results of FGF21 levels also agree with this fact. The interaction between energy metabolism and stress responses can be mediated by FGF21 and its overexpression due to mitochondrial dysfunction extends mice lifespan^20^. However, the liver of *Coq9*^*Q95X*^ mice also fail to show that FGF21 is the cause of the survival increase and body weight reduction in this mouse model.

Overall, the results of this study suggest that the effects of the low levels of COQ7 over the longevity on *Coq9*^*Q95X*^ mice could not be initiated at the mitochondria. An alternative option to explain those effects is the function of COQ7 in the nucleus. Some studies have recently stated that, under certain conditions, mitochondrial proteins could be found in the nucleus to mediate a mitochondrial to nuclear retrograde signalling^31^. One of these proteins is COQ7, which has a nuclear targeting sequence, so could be located either in the nucleus or in the mitochondria. In the nucleus, COQ7 suppresses the mitochondrial unfolded protein response (UPR^mt^), suggesting that it acts to prevent activation of this pathway during non-stress conditions^19^. Therefore, an overall reduction in the levels of COQ7 would reduce also its location in the nucleus and, as a consequence, the UPR^mt^ would be activated, promoting longevity by mechanisms of proteostais^19,32^. Nevertheless, the lack of changes in the levels of GRP75 and CLPP suggest that UPR^mt^ is neither the mechanism of the increased survival in *Coq9*^*Q95X*^ mice. This data may also question the location of COQ7 into the nucleus, as it has been recently stated^33^.

Even if we have not identified a clear mechanism in the hepatic mitochondria to explain the increased longevity in *Coq9*^*Q95X*^ mice, we have to take into account that this study is limited to the liver and, thus, other tissues were not evaluated. We focused on the liver to compare our data with those in the *Coq7*^+/−^ mice. Lapointe and colleagues reported a mild mitochondrial respiratory chain dysfunction in the liver from 3-months-old *Coq7*^+/−^ mice^13^. As the authors state, those changes provide protection from the age-dependent loss of mitochondrial function and attenuation of oxidative stress in the liver from 12 and 23-months-old *Coq7*^+/−^ mice. In the *Coq9*^*Q95X*^ mouse model, we do not detect significant differences in mitochondrial bioenergetics and oxidative stress markers at any age^34^. In any case, we cannot exclude that the target cells and tissues for promoting longevity in the *Coq7*^+/−^ and *Coq9*^*Q95X*^ mouse models are different. Therefore, the effects on the body weight and survival in *Coq9*^*Q95X*^ mice could be due to the changes in other tissues in which the CoQ levels are reduced, such as skeletal muscle, kidney or brain^16^. Similarly, additional mechanisms in tissues different from the liver could contribute to the increased survival in *Coq7*^+/−^ mice^11^. Furthermore, we cannot either exclude that the increased longevity in *Coq9*^*Q95X*^ is due to the reduction in the levels of other CoQ biosynthetic proteins in the liver or other tissues, e.g. COQ5, COQ6 or COQ9^16^; even if this interpretation is based in a mere correlation and not in a direct evidence. Nevertheless, this interpretation would be supported by the fact that silencing of *Coq1*, *Coq2*, *Coq3*, *Coq4*, *Coq5*, *Coq6*, *Coq7* or *Coq8* extended the lifespan in *C. Elegans*^35^.

In conclusion, our study corroborates that low levels of CoQ biosynthetic proteins promote an increase of longevity. However, our results do not support the hepatic mitochondrial dysfunction and increased oxidative stress as the mechanism by which subphysiological levels of CoQ biosynthetic proteins induce an increase in lifespan. Therefore, these effects could be mediated by the mitochondria in other tissues or by other unknown mechanisms. Further studies about CoQ biosynthetic proteins would be important to elucidate the role of these proteins in aging.

## Methods

### Mouse model

The *Coq9*^*R239X*^ mouse model was previously generated and characterized under a mix of C57BL/6N and C57BL/6J genetic background^16^. *Coq9*^*Q95X*/+^ mice were crossbreed in order to generate *Coq9*^+/+^*, Coq9*^*Q95X*/+^, *and Coq9*^*Q95X*/*Q95X*^ (referred in the article to as *Coq9*^*Q95X*^).

Animals were genotyped and assigned in experimental groups base on their age (young adult between 6 and 7 months of age, middle-age between 10 and 16 months of age and old with more than 22 months of age), and only homozygous wild-type and mutant mice were used in the study. Data were randomly collected and processed as well.

Mice were housed in the Animal Facility of the University of Granada under an SPF zone with lights on at 7:00 AM and off at 7:00 PM. Mice had unlimited access to water and rodent chow (2914 Teklad global 14% protein rodent maintenance diet). All experiments were performed according to a protocol approved by the Institutional Animal Care and Use Committee of the University of Granada (procedures 92-CEEA-OH-2015) and were in accordance with the European Convention for the Protection of Vertebrate Animals used for Experimental and Other Scientific Purposes (CETS #123), the directive 2010/63/EU on the protection of animals used for scientific purposes and the Spanish law (R.D. 53/2013).

Lifespan was determined by recording the age of spontaneous death, or when one of the following criteria was met: unresponsiveness to touch, slow respiration, coldness to touch, a hunched up position with matted fur, or sudden weight loss. Mice were weighed at 3, 6, 12 and 20 months of age.

### Genotyping

DNA was extracted from the mice tail tips, and PCR of *Coq9* gene was performed as described in Supplementary Material. To amplify wt allele, we used the following primers: forward, CTGGGAACTGAGCTCAGATCTTCTAC; reverse, GTGTCCAGGGATTTGAGTTCTTATGC. To amplify mutant allele, we used the following primers: forward, GAGATGGCGCAACGCAATTAAT; reverse, ATGAGAGATAGAGAGAGGCGGAGAGG^16^.

### Quantification of CoQ9 levels

After lipid extraction from homogenized tissues CoQ_9_ levels were determined via reversed-phase HPLC coupled to electrochemical (EC) detection^16^. The results were expressed in ng CoQ_9_/mg prot.

### Sample preparation and Western blot analysis

For Western blot analyses in liver and muscle, samples were homogenized in T-PER® buffer (Thermo Scientific) with protease inhibitor cocktail (Pierce) at 1,100 rpm in a glass–Teflon homogenizer. Homogenates were sonicated and centrifuged at 1,000 g for 5 min at 4 °C, and the resultant supernatants were used for Western blot analysis.50 μg of protein from the sample extracts was electrophoresed in 4–15% Mini-PROTEAN® TGX™ precast gels (Bio-Rad) using the electrophoresis mini-PROTEAN® Tetra Cell System (Bio-Rad). Proteins were transferred onto PVDF 0.45 μm membranes using a mini Trans-Blot® Turbo™Transfer System (Bio-Rad) and probed with target antibodies. Protein–antibody interactions were detected with peroxidase-conjugated horse anti-mouse or anti-rabbit IgG antibodies using Amersham^TM^ ECL^TM^ Prime Western Blotting Detection Reagent (GE Healthcare, Buckinghamshire, UK). Band quantification was carried out using an Image Station 2000R (Kodak, Spain) and a Kodak 1D 3.6 software. Protein band intensity was normalized to Tom20 (mitochondrial proteins) or GAPDH (citosolic proteins), and the data expressed in terms of percent relative to wild-type mice^36^.

The following primary antibodies were used: anti-COQ7 (Proteintech^TM^, 15083-1-AP), anti-COQ6 (Santa Cruz Biotechnology, sc-393932), anti-COQ5 (Proteintech™, 17453-1-AP), anti-CABC1 (COQ8A) (Abnova, M04A), anti-SOD2 (MnSOD) (Proteintech™, 24127-1-AP), anti-GPx-1/2 (Santa Cruz Biotechnology, sc-30147), anti-GRd (Santa Cruz Biotechnology, sc-32886), anti-UCP3 (Proteintech^TM^, 10750-1-AP), anti-UCP2 (Proteintech^TM^, 11081-1-AP), anti-FGF21 (Abcam, ab171941) anti-Tom20 (Proteintech^TM^, 11802-1-AP), anti-GAPDH (Santa Cruz Biotechnology, sc-166574).

### Respiratory chain activities

Liver samples were homogenated in CPT medium (0.05 M Tris-HCl, 0.15 M KCl, pH 7.5) at 1,100 rpm in a glass–Teflon homogenizer. Homogenates were sonicated and centrifuged at 600 g for 20 min at 4 °C, and the resultant supernatants were used to measure respiratory chain activities (CI + III, CII + III, CI, CII, CIII and CIV) as described elsewhere^16,17,37^. The results were expressed in nmol reduced cyt c/min/mg prot. To correct the results by possible changes in mitochondrial mass, the results were also normalized by the activity of Citrate Synthase (CS).

### Immunohistochemistry

Liver were formalin-fixed and paraffin-embedded. Multiple sections (4 μm) were deparaffinized with xylene. Immunohistochemistry was carried out using anti-8OHdG (QED Bioscience, 12501) as primary antibody^17^. Dako Animal Research Kit for mouse primary antibodies (Dako Diagnóstico S.A., Spain) was used for the qualitative identification of antigens by light microscopy. Sections were examined at 10× magnifications with a ZEISS Primo Star microscope, and the images were scanned under equal light conditions with the Axio Vision 4.8.2 computer program. The 8OHdG positive signal was quantified by using the software ImageJ (National Institutes of Health, USA) and the results were expressed by the percentage of the positive signal.

### Statistical analysis

All statistical analyses were performed using the GraphPad Prism version 6.00 for Windows, GraphPad Software (La Jolla CA USA). Data are expressed as the mean ± SD of three–six experiments per group. Studies with two experimental groups were evaluated using multiple Student’s *t-*test one per row. A *P-*value of <0.05 was considered to be statistically significant.

Survival was graphed by the Kaplan-Meier method and analyzed by the log-rank (Mantel-Cox) test and the Gehan-Breslow-Wilcoxon test.

## Electronic supplementary material

Supplementary figures

Full-length gels and blot
