# Supplementary material for: Reduction in the levels of CoQ biosynthetic proteins is related to an increase in lifespan without evidence of hepatic mitohormesis
Source: Sci Rep. 2018 Sep 18;8:14013. doi: 10.1038/s41598-018-32190-y (PMC6143522; doi:10.1038/s41598-018-32190-y)
Supplement: Supplementary file 1 — Supplementary figures [file 41598_2018_32190_MOESM1_ESM.docx]

Reduction in the levels of CoQ biosynthetic proteins is related to an increase in lifespan without evidence of hepatic mitohormesis

María Rodríguez-Hidalgo^1^, Marta Luna-Sánchez^1^, Agustín Hidalgo-Gutiérrez^1^, Eliana Barriocanal-Casado^1^, Cristina Mascaraque^1^, Darío-Acuña-Castoviejo^1,2^, Margarita Rivera^3^, Germaine Escames^1,2^, Luis C. López ^1,2,^*

^1^ Institute of Biotechnology, Biomedical Research Centre and Department of Physiology, Faculty of Medicine; University of Granada, Spain.

^2^ Centro de Investigación Biomédica en Red Fragilidad y Envejecimiento Saludable (CIBERFES), Spain.

^3^ Institute of Neurosciences, Biomedical Research Centre and Biochemistry and Molecular Biology II; University of Granada, Spain.

***** Email: [luisca@ugr.es](mailto:luisca@ugr.es)

**Supplementary figures**

**
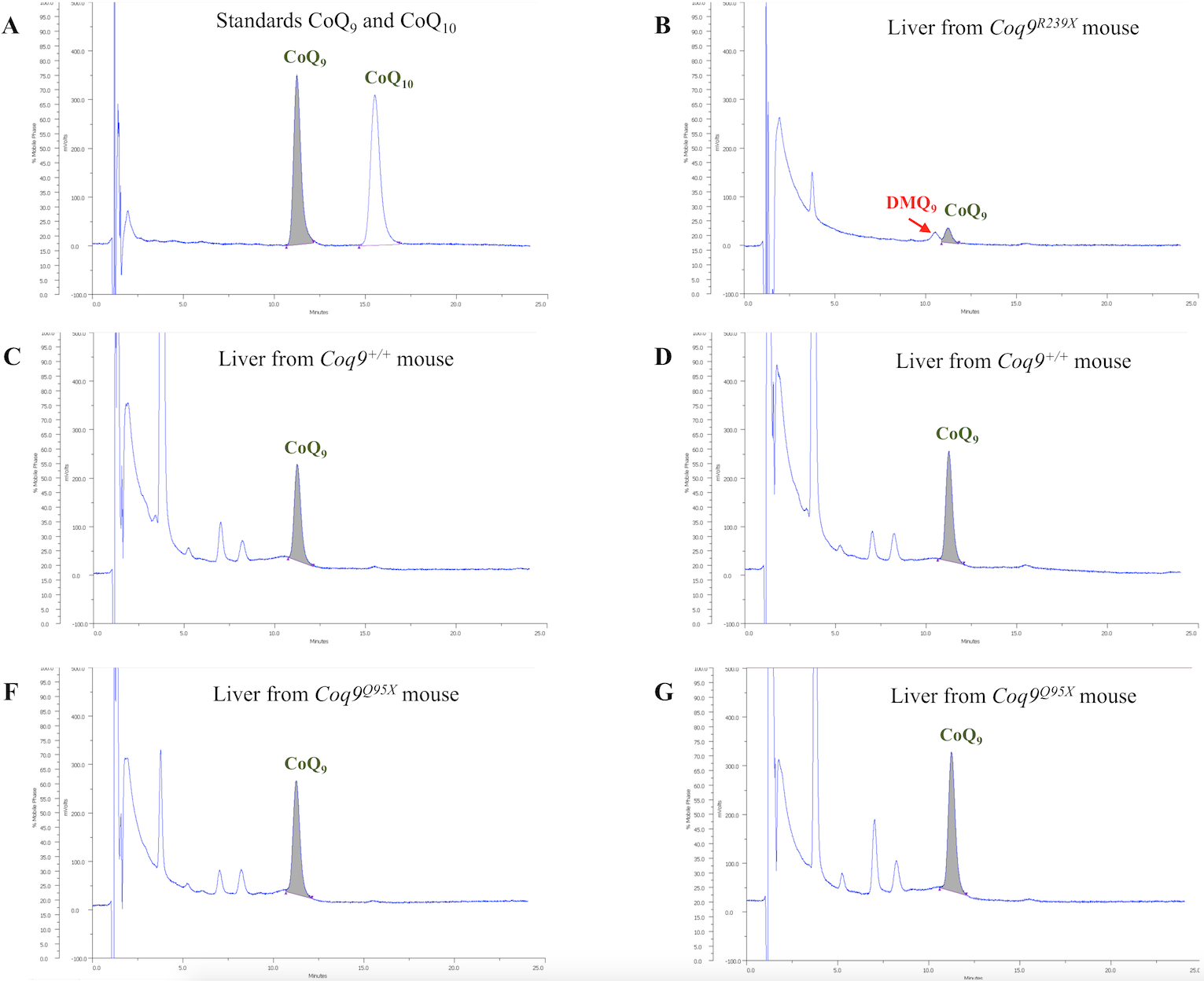
**

**Figure S1. HPLC chromatographs for the detection of Quinones.**

1. Standards of CoQ_9_ and CoQ_10_ at a concentration of 300 ng/ml of each compund.

(B-G) Indentification of CoQ_9_ and DMQ_9_ in liver extracts from *Coq9^R239X^* (B), *Coq9^+/+^* (C-D) and *Coq9^Q95X^* (F-G) mice at 6 months of age. The chromatograph of the liver extract from a *Coq9^R239X^* mouse was used as a possitive control for the detection of DMQ_9_.

**
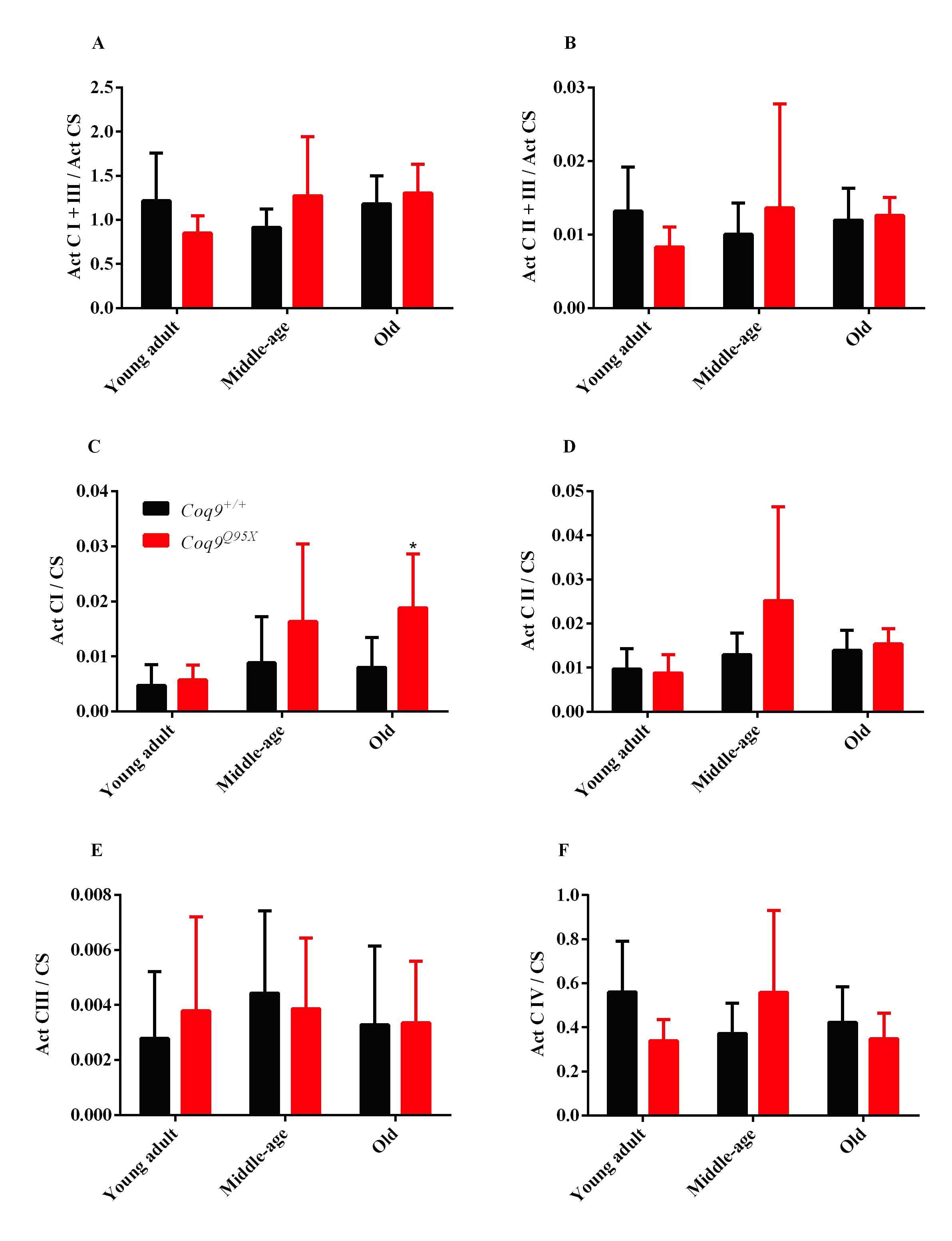
**

**Figure S2. Mitochondrial respiratory chain activities normalized by Citrate Synthase activity as a marker of mitochondrial mass.**

A–C. CI+III (A), CII+III (B), CI (C), CII (D), CIII (E) and CIV (F) activities normalized by Citrate Synthase Activity (CS) in liver homogenates of young adult, middle-age and old *Coq9^Q95X^* and *Coq9*^+/+^ male mice. **P*< 0.05; *Coq9^Q95X^* versus *Coq9*^+/+^ littermates mice.

Data information: (A–F) Data are expressed as mean ± SD. Statistical analyses were performed on *Coq9*^+/+^ male mice versus *Coq9^Q95X^*male mice by Multiple Student's *t-*test one per row. *Coq9*^+/+^ mice *n* = 6; *Coq9^Q95X^* mice *n* = 6, at each age.

**
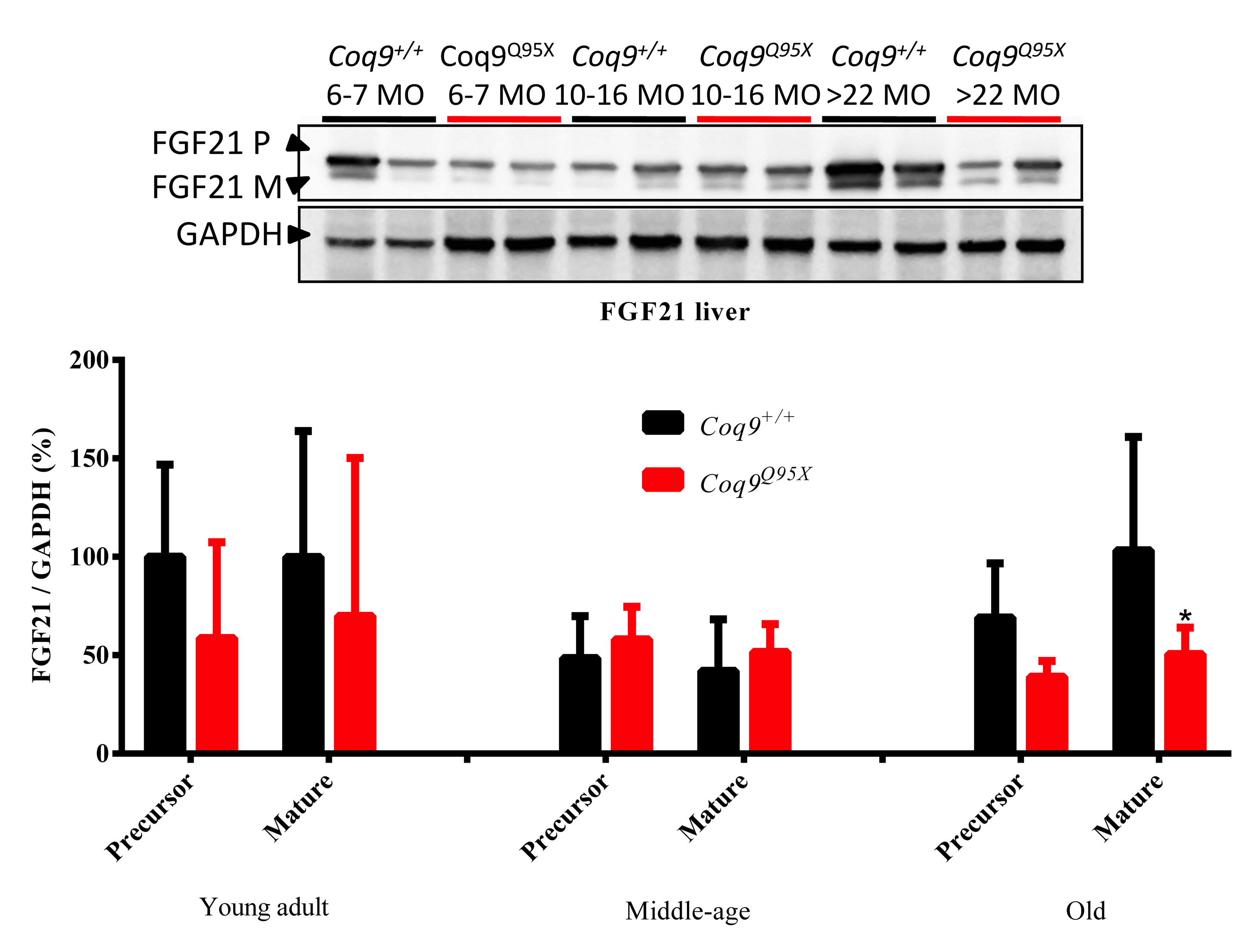
**

**Figure S3. Leves of FGF21.**

Representative western blot of FGF21 and their quantitative analysis in the liver of young adult, middle-age and old *Coq9^Q95X^* and *Coq9*^+/+^ male mice. **P*< 0.05; ***P*< 0.01; *** *P*< 0.001; *Coq9^Q95X^* versus *Coq9*^+/+^ mice. The cytosolic GAPDH was used as loading control. P = precursor; M = mature.

Data information: Data are expressed as mean ± SD. Statistical analyses were performed on *Coq9*^+/+^ male mice versus *Coq9^Q95X^* male mice by Multiple Student's *t-*test one per row. *Coq9*^+/+^ mice *n* = 6; *Coq9^Q95X^* mice *n* = 6, at each age.

**
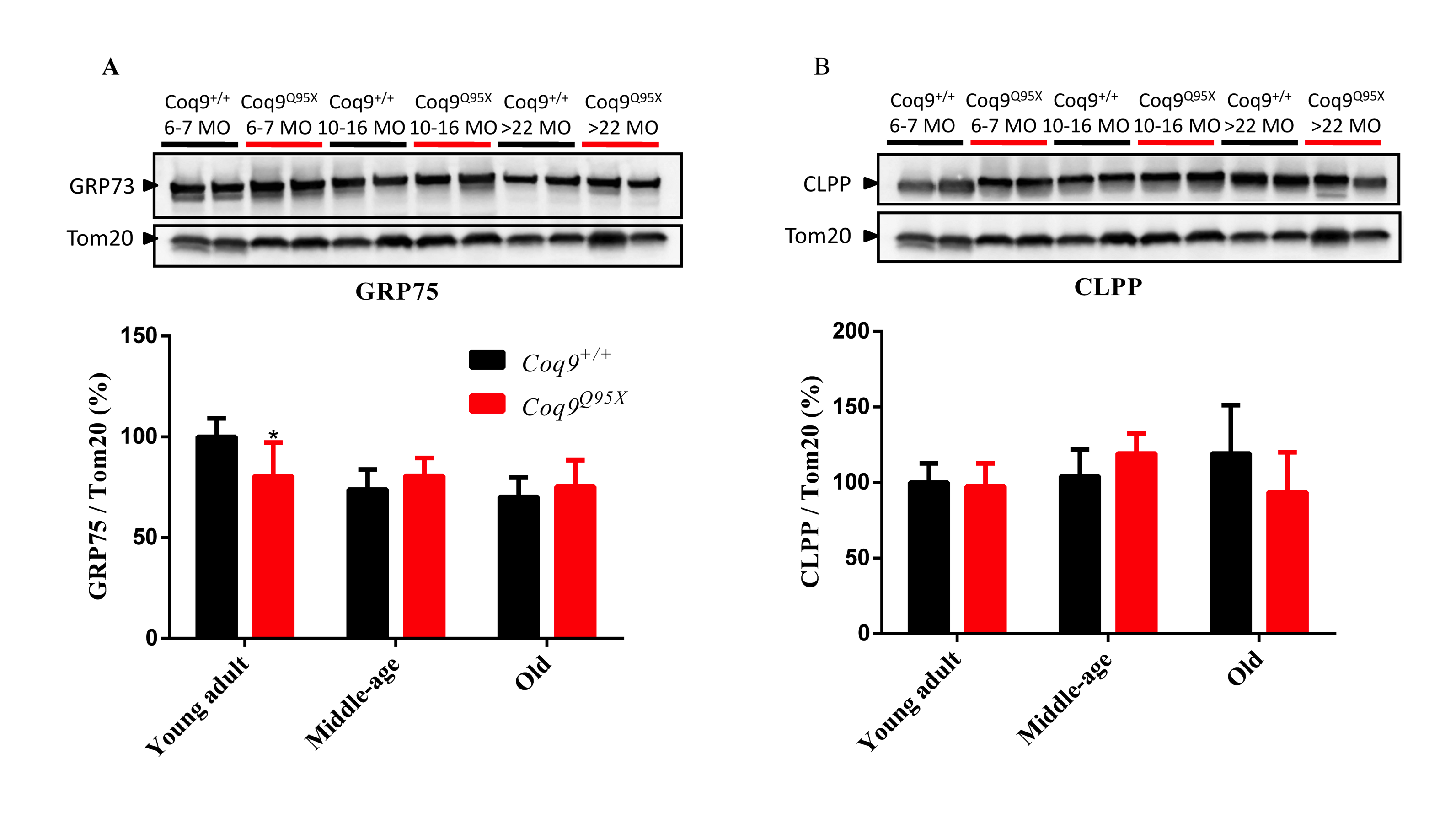
**

**Figure S4. Levels of proteins involved in the mitochondrial stress responses.**

A and B. Representative western blot of GRP75 (A) and CLPP (B) their quantitative analysis in the liver of young adult, middle-age and old *Coq9^Q95X^* and *Coq9*^+/+^ male mice. **P*< 0.05; ***P*< 0.01; *** *P*< 0.001; *Coq9^Q95X^* versus *Coq9*^+/+^ littermates mice. The mitochondrial Tom20 was used as loading control.

Data information: (A-B) Data are expressed as mean ± SD. Statistical analyses were performed on *Coq9*^+/+^ male mice versus *Coq9^Q95X^* male mice by Multiple Student's *t-*test one per row. *Coq9*^+/+^ mice *n* = 6; *Coq9^Q95X^* mice *n* = 6, at each age.
