# Supplementary material for: Reduction in the levels of CoQ biosynthetic proteins is related to an increase in lifespan without evidence of hepatic mitohormesis
Source: Sci Rep. 2018 Sep 18;8:14013. doi: 10.1038/s41598-018-32190-y (PMC6143522; doi:10.1038/s41598-018-32190-y)
Supplement: Supplementary file 2 — Full-length gels and blot [file 41598_2018_32190_MOESM2_ESM.docx]

Reduction in the levels of CoQ biosynthetic proteins is related to an increase in lifespan without evidence of hepatic mitohormesis

María Rodríguez-Hidalgo^1^, Marta Luna-Sánchez^1^, Agustín Hidalgo-Gutiérrez^1^, Eliana Barriocanal-Casado^1^, Cristina Mascaraque^1^, Darío-Acuña-Castoviejo^1,2^, Margarita Rivera^3^, Germaine Escames^1,2^, Luis C. López ^1,2,^*

^1^ Institute of Biotechnology, Biomedical Research Centre and Department of Physiology, Faculty of Medicine; University of Granada, Spain.

^2^ Centro de Investigación Biomédica en Red Fragilidad y Envejecimiento Saludable (CIBERFES), Spain.

^3^ Institute of Neurosciences, Biomedical Research Centre and Biochemistry and Molecular Biology II; University of Granada, Spain.

***** Email: [luisca@ugr.es](mailto:luisca@ugr.es)

**Full-length gels and blots**

Figure 2B. Levels of CoQ and the biosynthetic proteins COQ7, COQ5, COQ6 and COQ8A.

Coq9^Q95X^

10-16 MO

Coq9^+/+^

>22 MO

Coq9^Q95X^

>22 MO

Coq9^+/+^

10-16 MO

Coq9^Q95X^

6-7 MO

Coq9^+/+^

6-7 MO


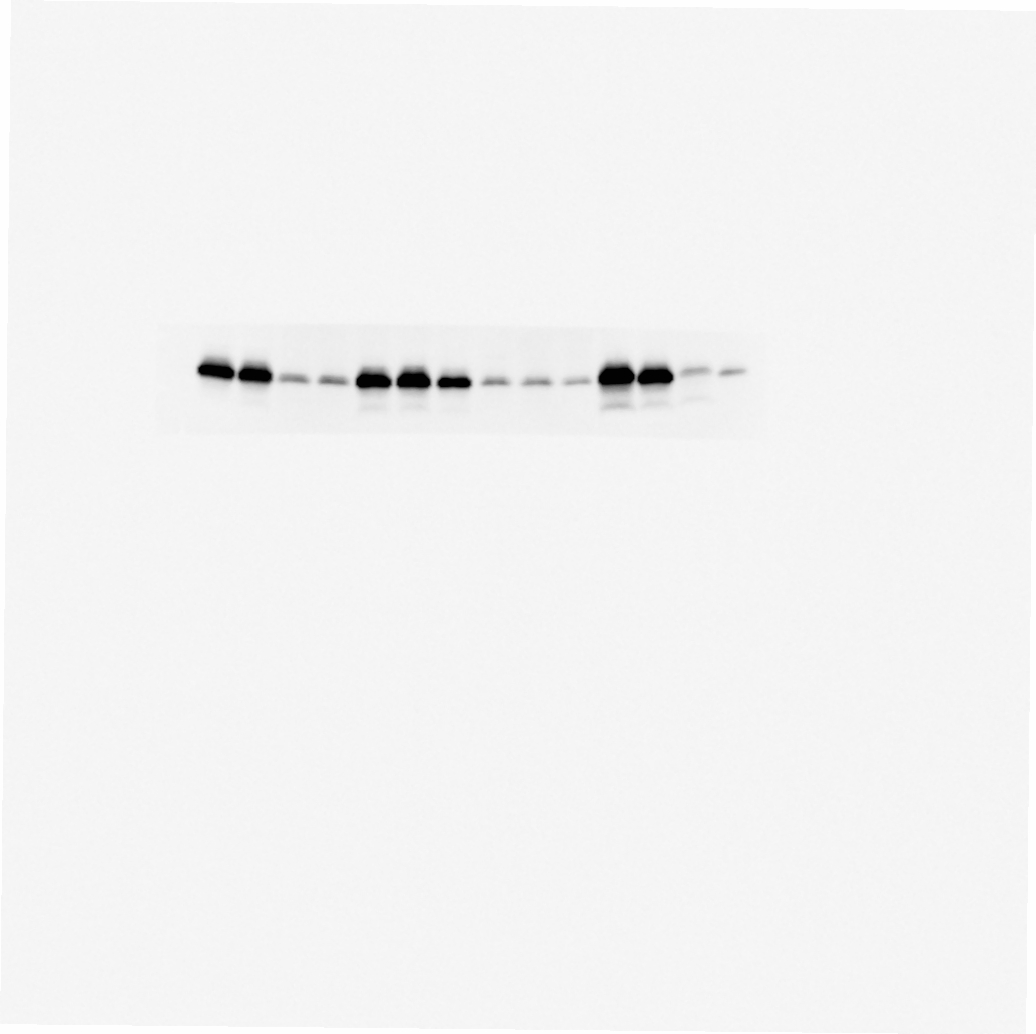


25 kDa -

15 kDa -

Spectra™ Multicolor Broad Range Protein Ladder Thermo Scientific™

COQ7 (23 kDa)

Coq9^Q95X^

>22 MO

Coq9^Q95X^

10-16 MO

Coq9^Q95X^

6-7 MO

Coq9^+/+^

>22 MO

Coq9^+/+^

10-16 MO

Coq9^+/+^

6-7 MO


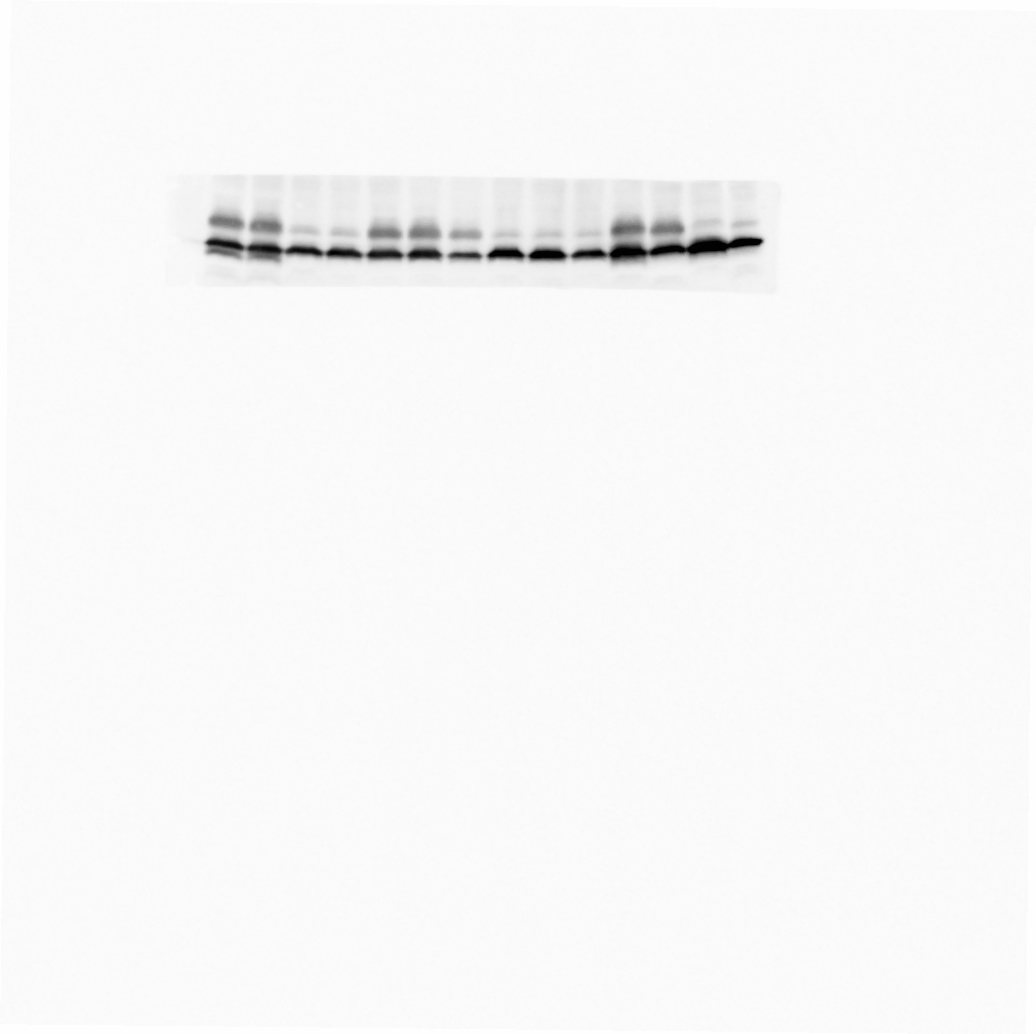


25 kDa -

15 kDa -

Post-stripping membrane

Tom20 (16 kDa)

Note: lines 2, 3, 4, 5, 6, 7, 10, 11, 12, 13, 14, 15 in Figure 2B in the main text.

Figure 2C. Levels of CoQ and the biosynthetic proteins COQ7, COQ5, COQ6 and COQ8A.

COQ5 (tom)

Coq9^Q95X^

>22 MO

Coq9^+/+^

6-7 MO

Coq9^Q95X^

6-7 MO

Coq9^+/+^

10-16 MO

Coq9^+/+^

>22 MO

Coq9^Q95X^

10-16 MO


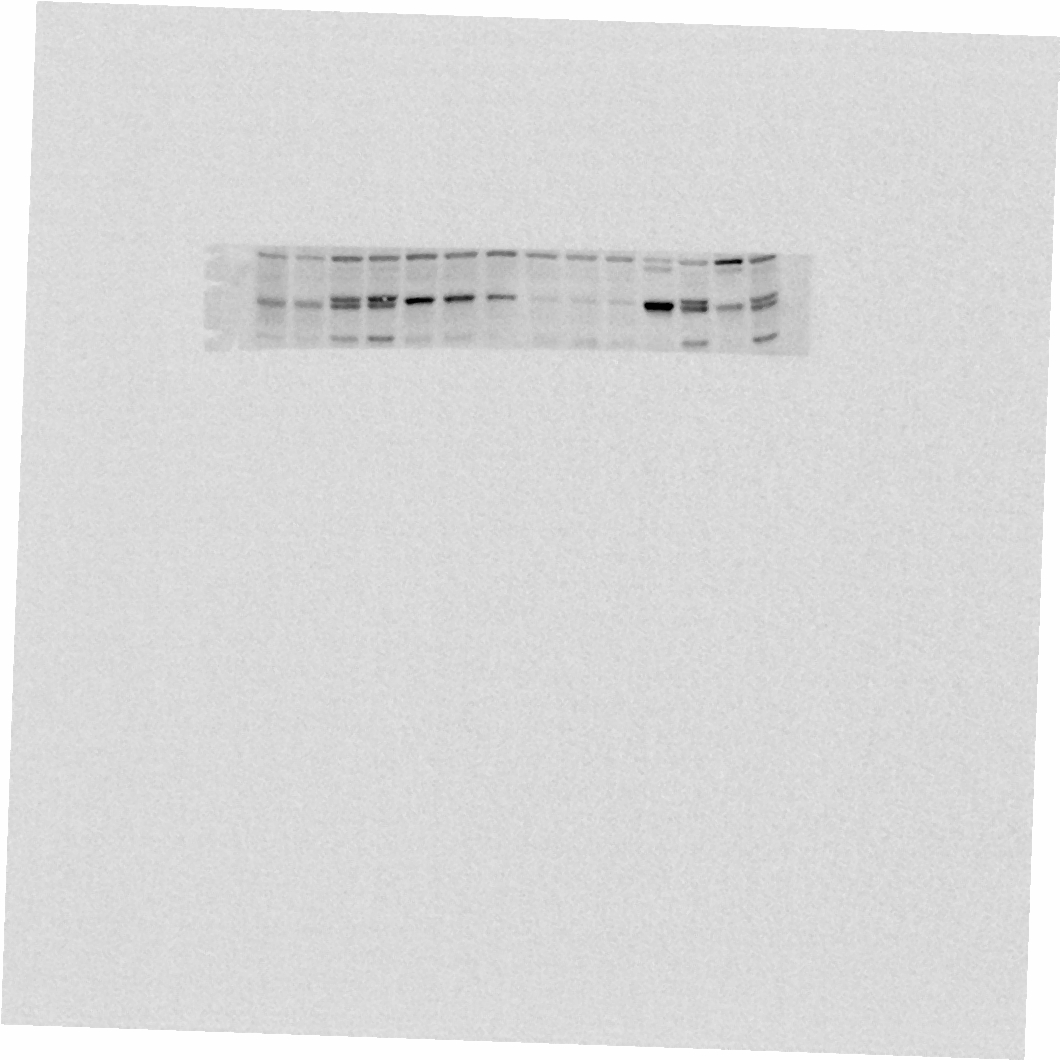


25 kDa -

35 kDa -

COQ5 (31.71 kDa)

Spectra™ Multicolor Broad Range Protein Ladder Thermo Scientific™

Tom20 (16 kDa)

Coq9^+/+^

>22 MO

Coq9^Q95X^

>22 MO

Coq9^Q95X^

10-16 MO

Coq9^Q95X^

6-7 MO

Coq9^+/+^

10-16 MO

Coq9^+/+^

6-7 MO


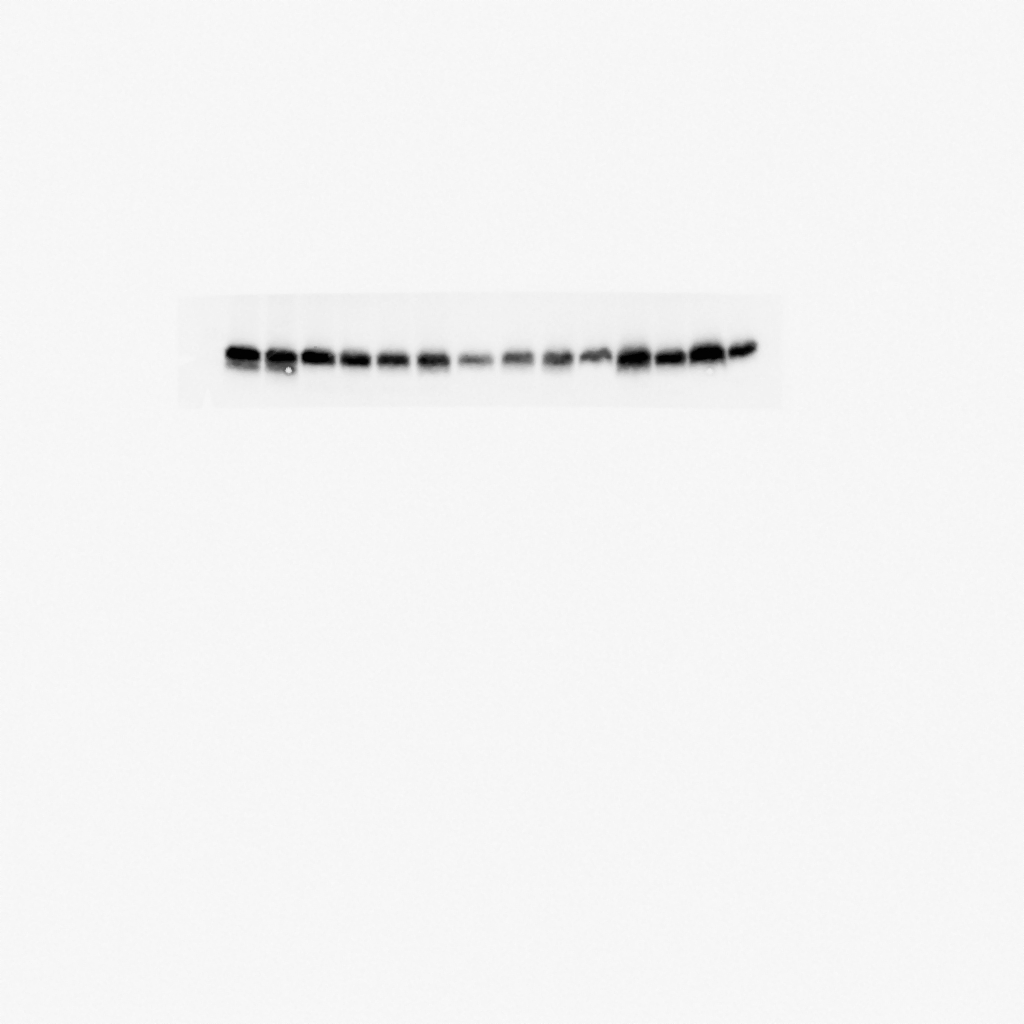


15 kDa -

Post-stripping membrane

Note: lines 2, 3, 4, 5, 6, 7, 10, 11, 12, 13, 14, 15 in Figure 2C in the main text.

Figure 2D. Levels of CoQ and the biosynthetic proteins COQ7, COQ5, COQ6 and COQ8A.

COQ6 (tom)

Coq9^Q95X^

6-7 MO

Coq9^+/+^

6-7 MO

Coq9^Q95X^

>22 MO

Coq9^Q95X^

10-16 MO

Coq9^+/+^

>22 MO

Coq9^+/+^

10-16 MO


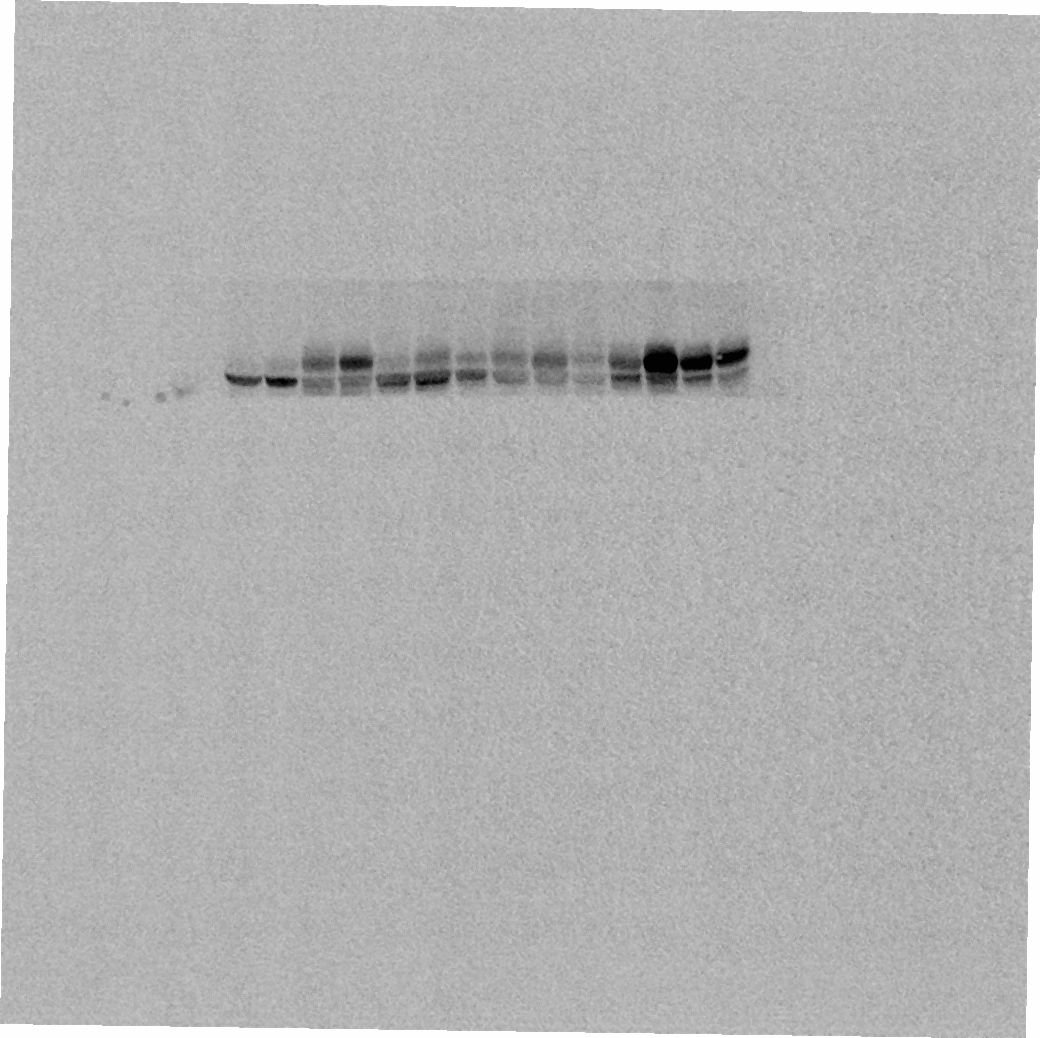


50 kDa -

COQ6 (47.66 kDa)

Spectra™ Multicolor Broad Range Protein Ladder Thermo Scientific™

Coq9^Q95X^

>22 MO

Coq9^+/+^

>22 MO

Coq9^Q95X^

10-16 MO

Coq9^Q95X^

6-7 MO

Coq9^+/+^

10-16 MO

Coq9^+/+^

6-7 MO


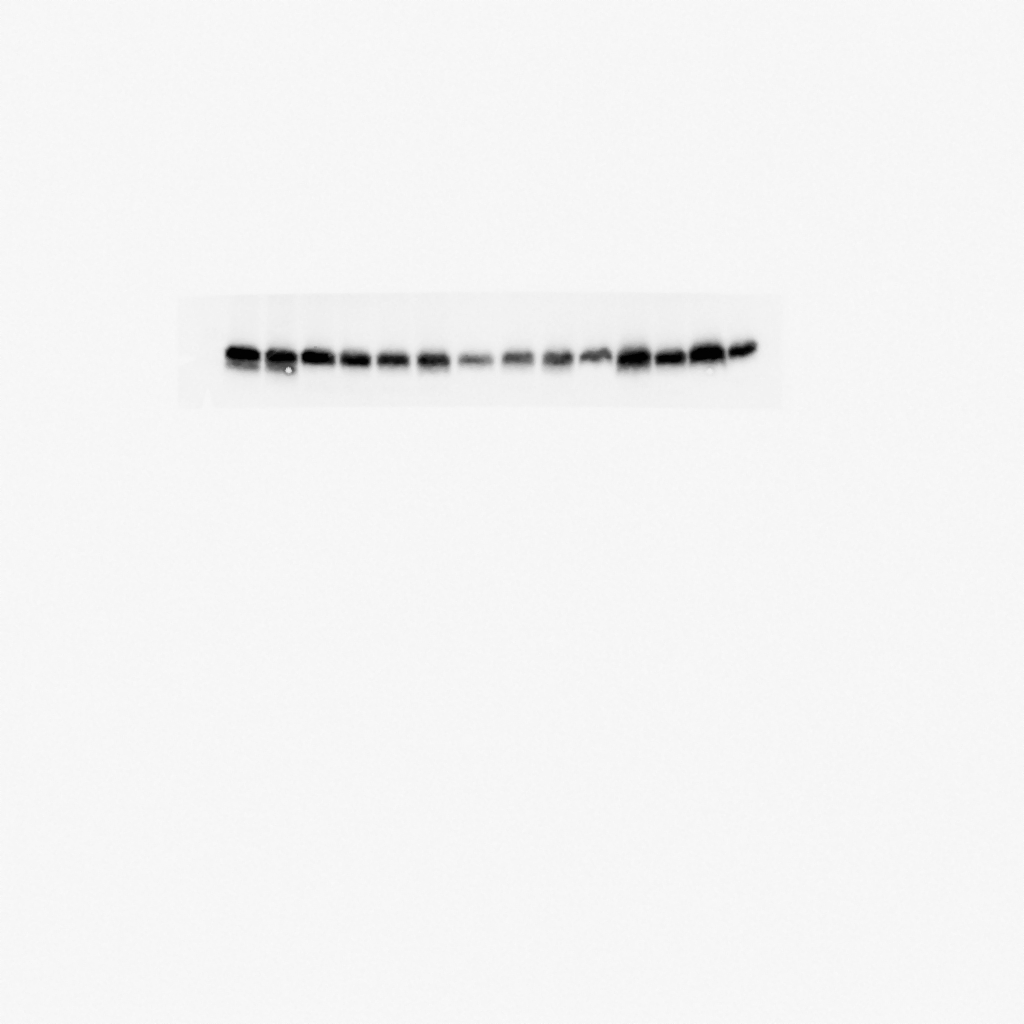


Post-stripping membrane

15 kDa -

Tom20 (16 kDa)

Note: lines 2, 3, 4, 5, 6, 7, 10, 11, 12, 13, 14, 15 in Figure 2D in the main text.

Figure 2E. Levels of CoQ and the biosynthetic proteins COQ7, COQ5, COQ6 and COQ8A.

COQ8A (tom)

Coq9^Q95X^

>22 MO

Coq9^Q95X^

10-16 MO

Coq9^Q95X^

6-7 MO

Coq9^+/+^

>22 MO

Coq9^+/+^

10-16 MO

Coq9^+/+^

6-7 MO


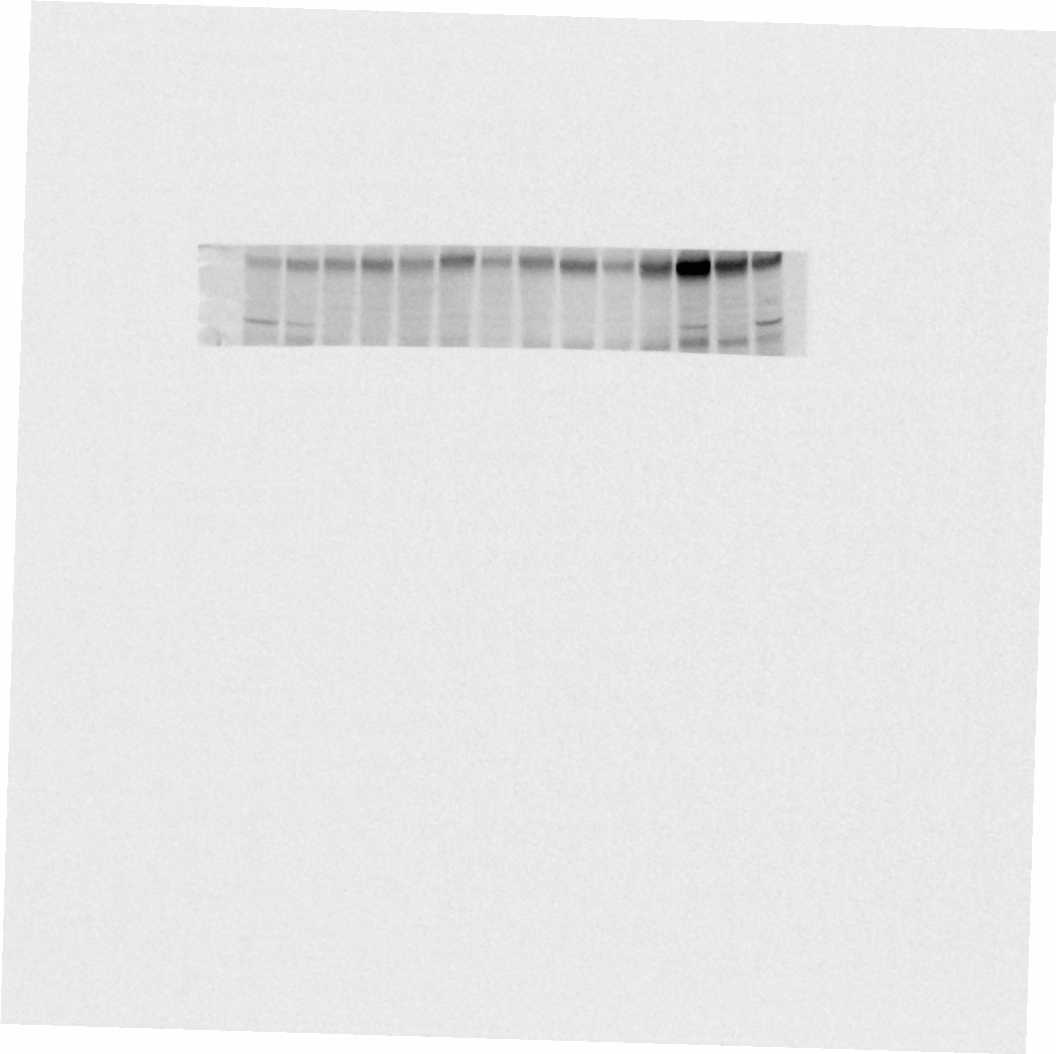


40 kDa -

50 kDa -

COQ8A (55.03 kDa)

Spectra™ Multicolor Broad Range Protein Ladder Thermo Scientific™

Coq9^+/+^

>22 MO

Coq9^Q95X^

>22 MO

Coq9^Q95X^

10-16 MO

Coq9^Q95X^

6-7 MO

Coq9^+/+^

10-16 MO

Coq9^+/+^

6-7 MO


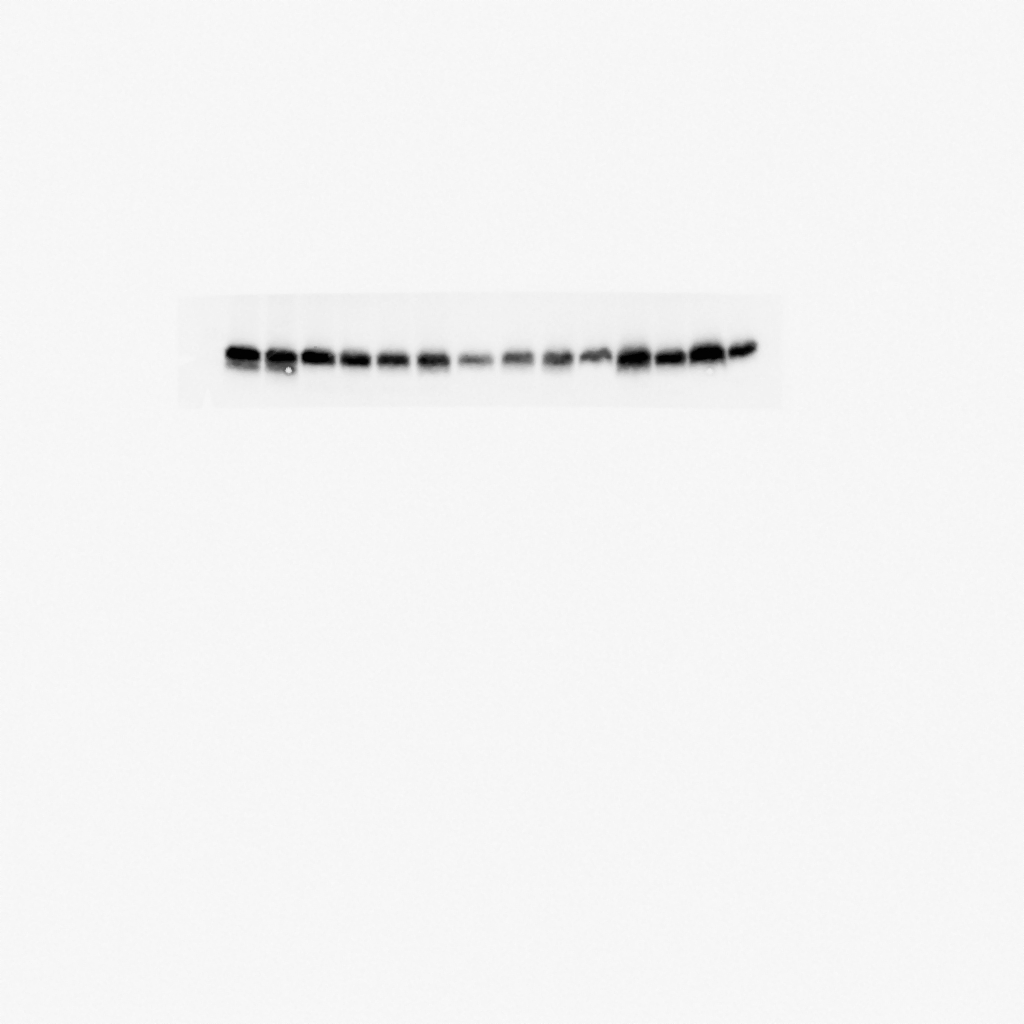


Post-stripping membrane

15 kDa -

Tom20 (16 kDa)

Note: lines 2, 3, 4, 5, 6, 7, 10, 11, 12, 13, 14, 15 in Figure 2E in the main text.

Figure 4A. Proteins involved in antioxidant systems.

MnSOD (tom)

Coq9^Q95X^

>22 MO

Coq9^+/+^

>22 MO

Coq9^Q95X^

10-16 MO

Coq9^+/+^

10-16 MO

Coq9^Q95X^

6-7 MO

Coq9^+/+^

6-7 MO


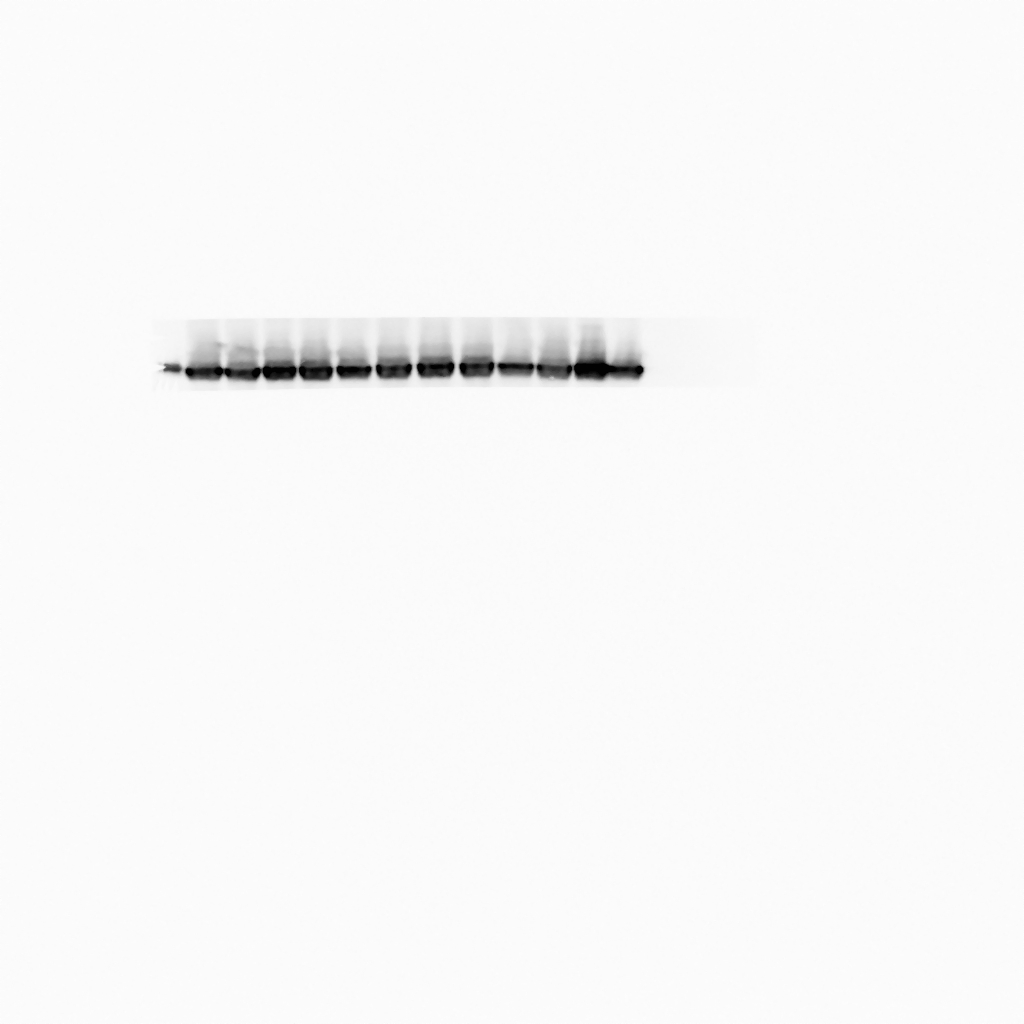


25 kDa -

Spectra™ Multicolor Broad Range Protein Ladder Thermo Scientific™

MnSOD (25 kDa)

Coq9^Q95X^

>22 MO

Coq9^Q95X^

10-16 MO

Coq9^Q95X^

6-7 MO

Coq9^+/+^

>22 MO

Coq9^+/+^

10-16 MO

Coq9^+/+^

6-7 MO


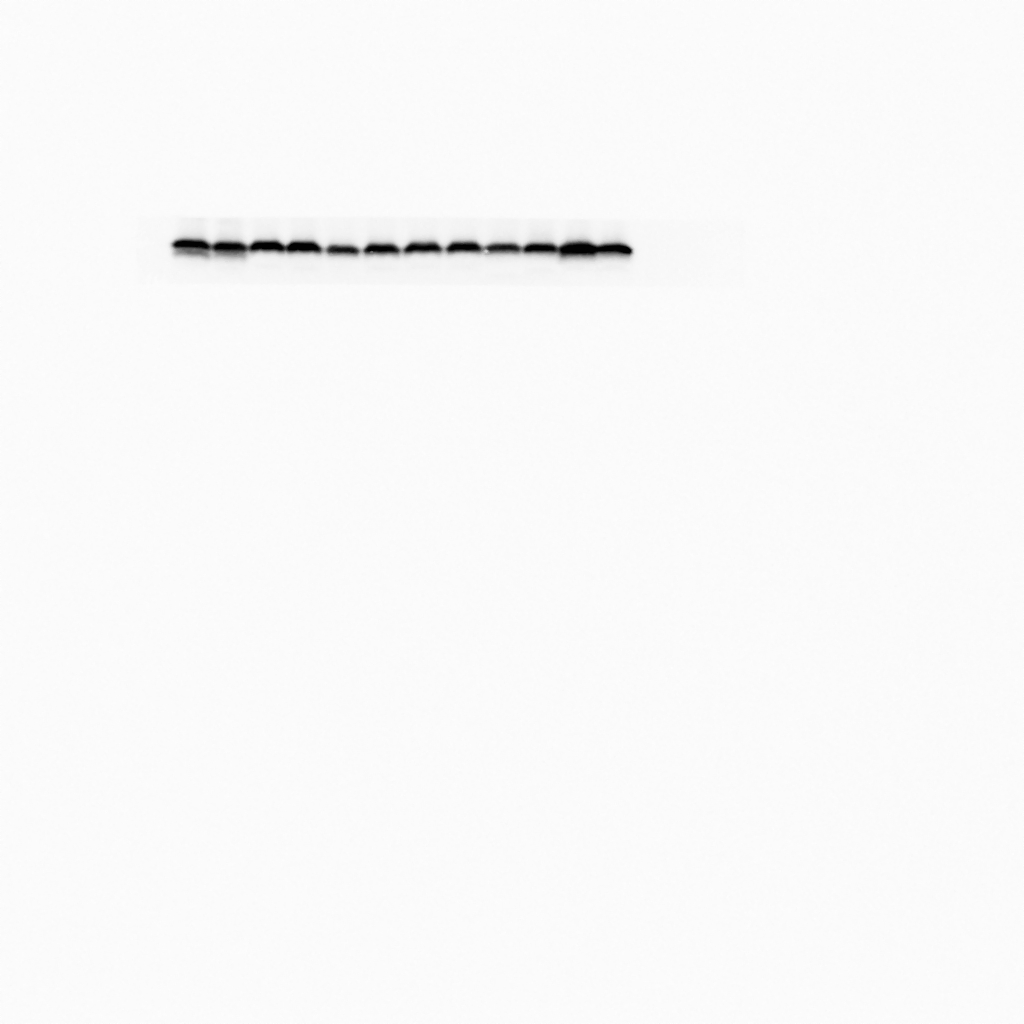


15 kDa -

Tom20 (16 kDa)

Note: lines 2, 3, 4, 5, 6, 7, 8, 9, 10, 11, 12, 13 in Figure 4A in the main text.

Figure 4B. Proteins involved in antioxidant systems.

Coq9^+/+^

6-7 MO

Coq9^+/+^

10-16 MO

Coq9^+/+^

>22 MO

Coq9^Q95X^

6-7 MO

Coq9^Q95X^

10-16 MO

Coq9^Q95X^

>22 MO


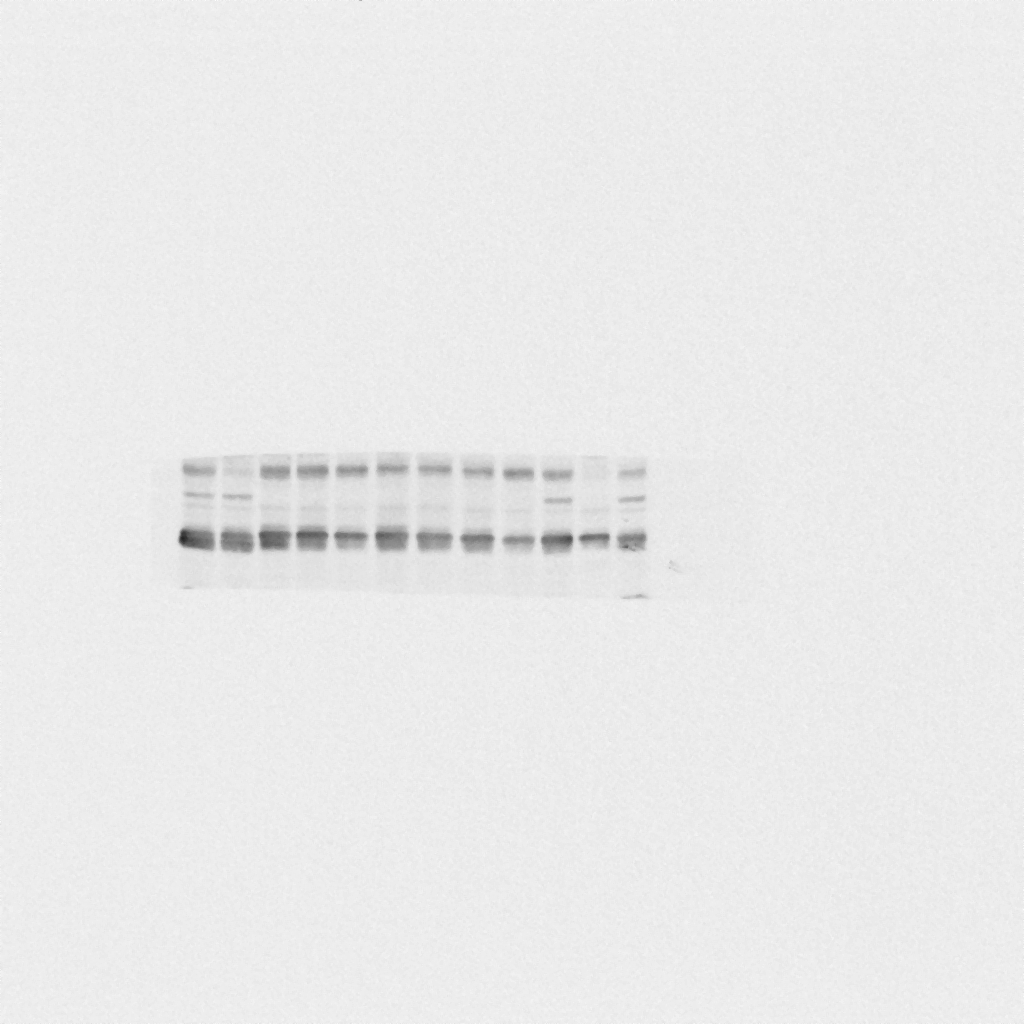


25 kDa -

35 kDa -

Spectra™ Multicolor Broad Range Protein Ladder Thermo Scientific™

GPx1/2 (23 kDa)

Coq9^+/+^

6-7 MO

Coq9^+/+^

10-16 MO

Coq9^+/+^

>22 MO

Coq9^Q95X^

6-7 MO

Coq9^Q95X^

10-16 MO

Coq9^Q95X^

>22 MO


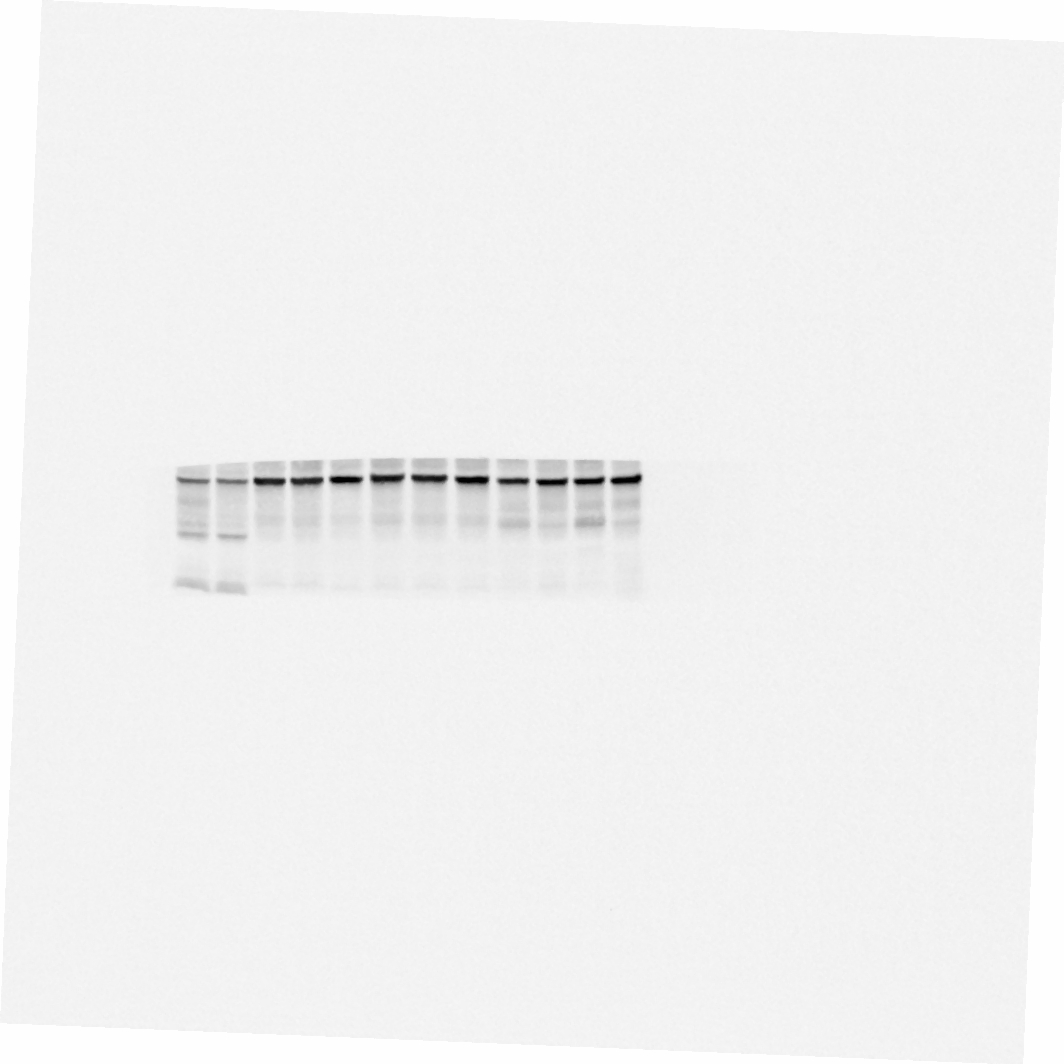


35 kDa -

25 kDa -

Post-stripping membrane

GAPDH (37 kDa)

Note: lines 2, 3, 4, 5, 6, 7, 8, 9, 10, 11, 12, 13 in Figure 4B in the main text.

Figure 4C. Proteins involved in antioxidant systems.

Coq9^Q95X^

>22 MO

Coq9^+/+^

6-7 MO

Coq9^Q95X^

10-16 MO

Coq9^Q95X^

6-7 MO

Coq9^+/+^

>22 MO

Coq9^+/+^

10-16 MO


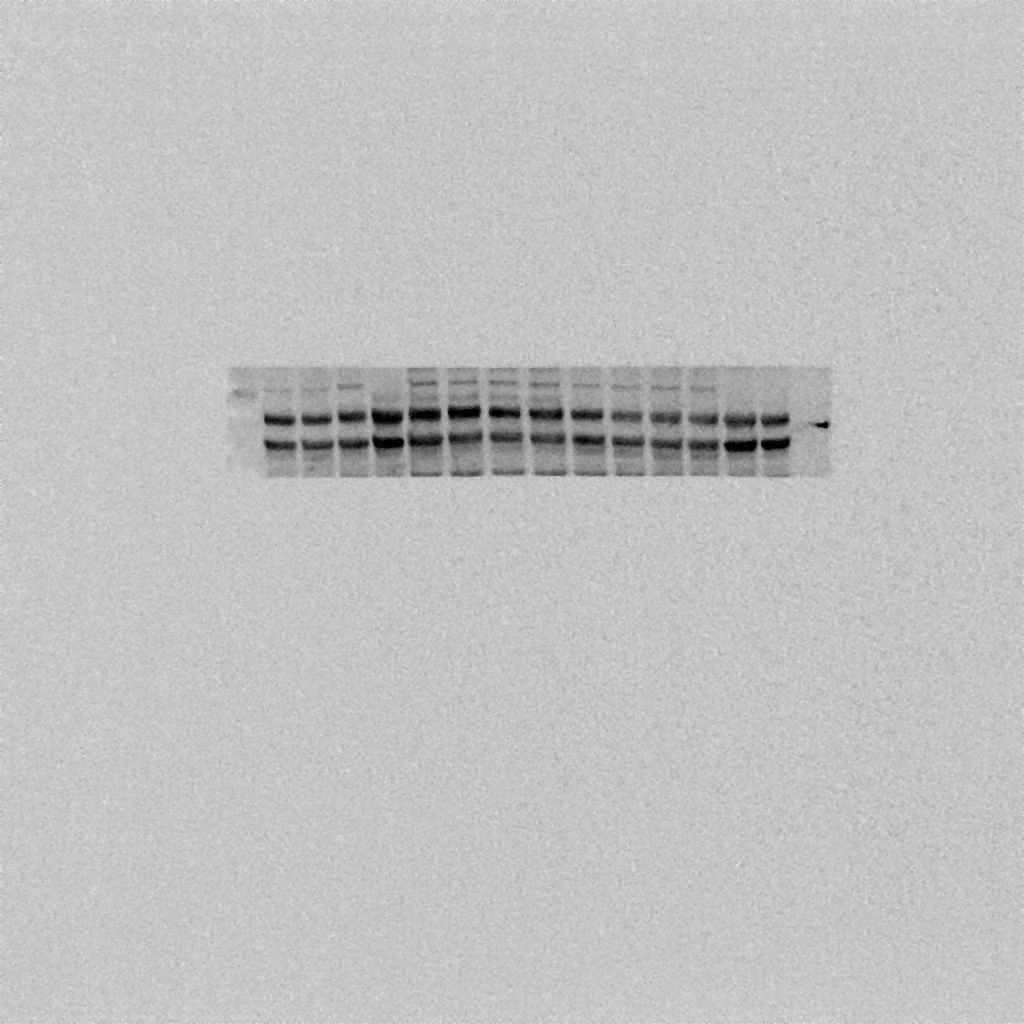


70 kDa -

50 kDa -

Spectra™ Multicolor Broad Range Protein Ladder Thermo Scientific™

GRd (50 kDa)

Coq9^Q95X^

>22 MO

Coq9^+/+^

6-7 MO

Coq9^Q95X^

10-16 MO

Coq9^Q95X^

6-7 MO

Coq9^+/+^

>22 MO

Coq9^+/+^

10-16 MO


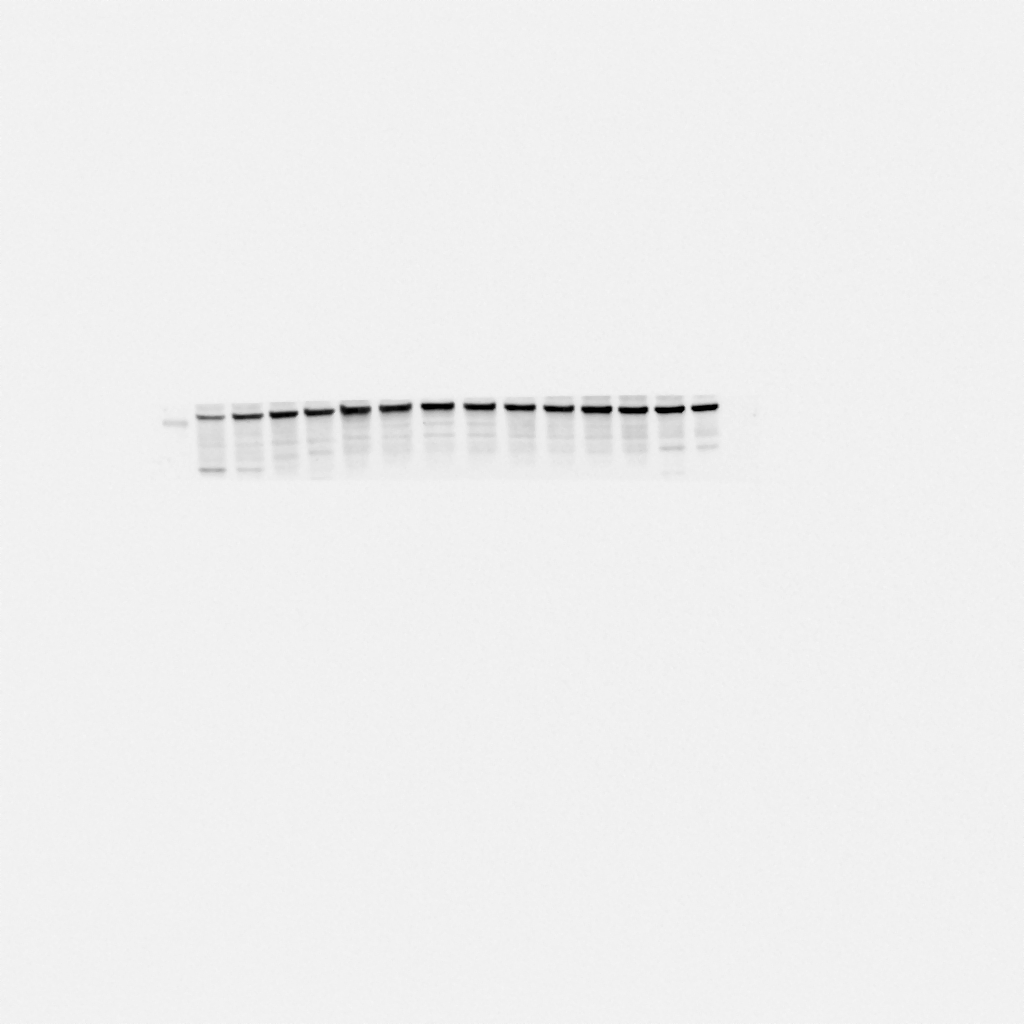


35 kDa -

GAPDH (37 kDa)

Note: lines 2, 3, 4, 5, 6, 7, 8, 9, 10, 11, 12, 13 in Figure 4C in the main text.

Figure 4D. Proteins involved in antioxidant systems.

Coq9^Q95X^

>22 MO

Coq9^Q95X^

10-16 MO

Coq9^Q95X^

6-7 MO

Coq9^+/+^

>22 MO

Coq9^+/+^

10-16 MO

Coq9^+/+^

6-7 MO


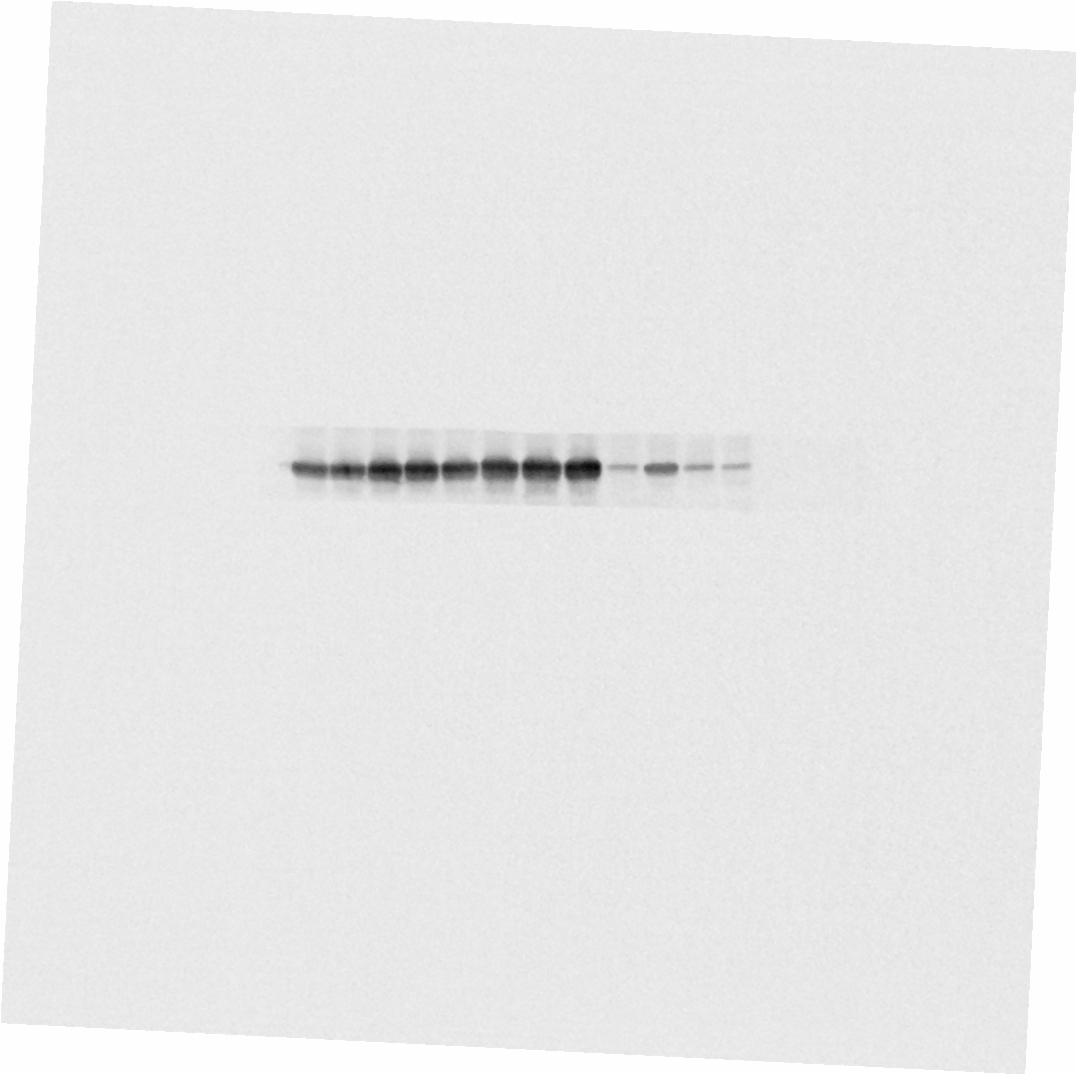


35 kDa -

Spectra™ Multicolor Broad Range Protein Ladder Thermo Scientific™

UCP2 (33 kDa)

Coq9^Q95X^

>22 MO

Coq9^Q95X^

10-16 MO

Coq9^Q95X^

6-7 MO

Coq9^+/+^

>22 MO

Coq9^+/+^

10-16 MO

Coq9^+/+^

6-7 MO


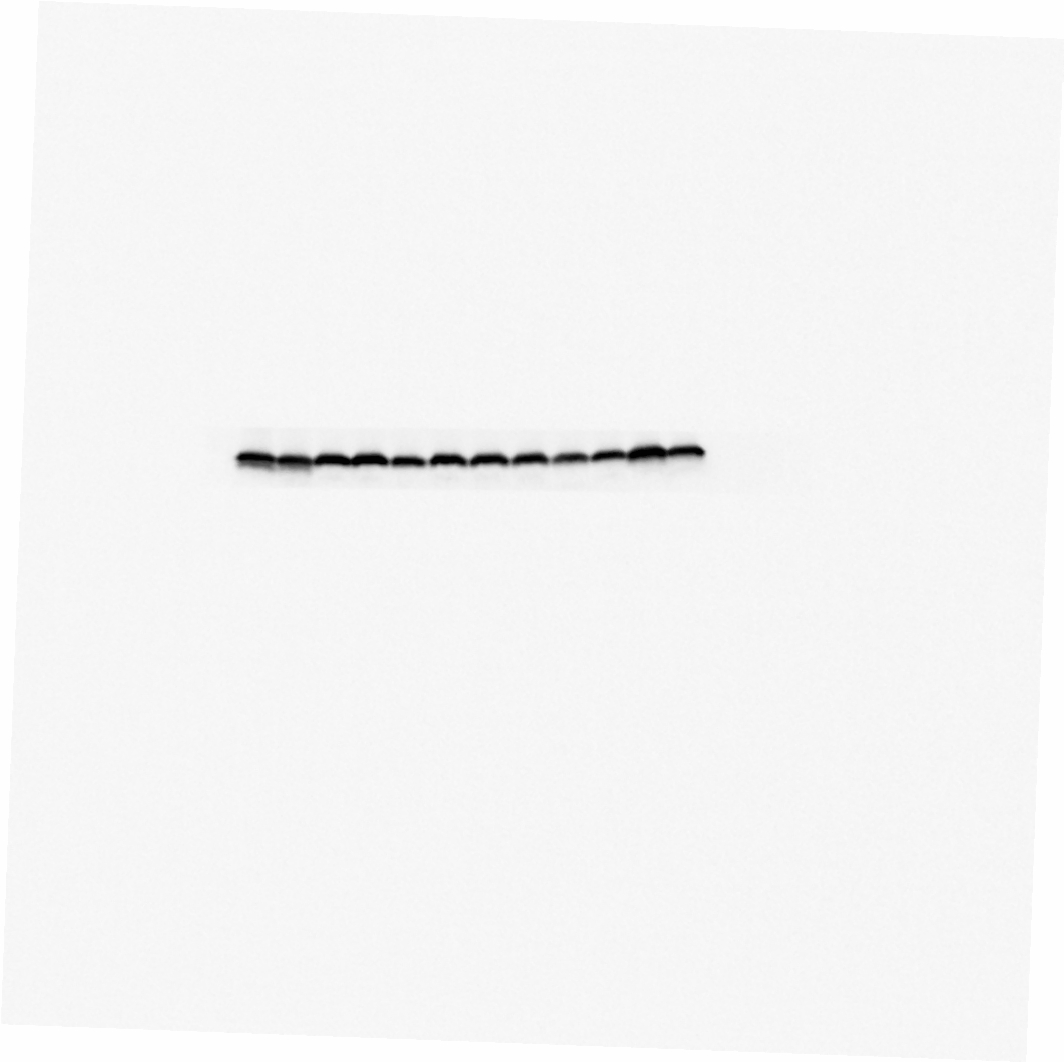


15 kDa -

Tom20 (16 kDa)

Note: lines 2, 3, 4, 5, 6, 7, 8, 9, 10, 11, 12, 13 in Figure 4D in the main text.

Figure 4E. Proteins involved in antioxidant systems.

Coq9^+/+^

6-7 MO

Coq9^+/+^

10-16 MO

Coq9^+/+^

>22 MO

Coq9^Q95X^

6-7 MO

Coq9^Q95X^

10-16 MO

Coq9^Q95X^

>22 MO


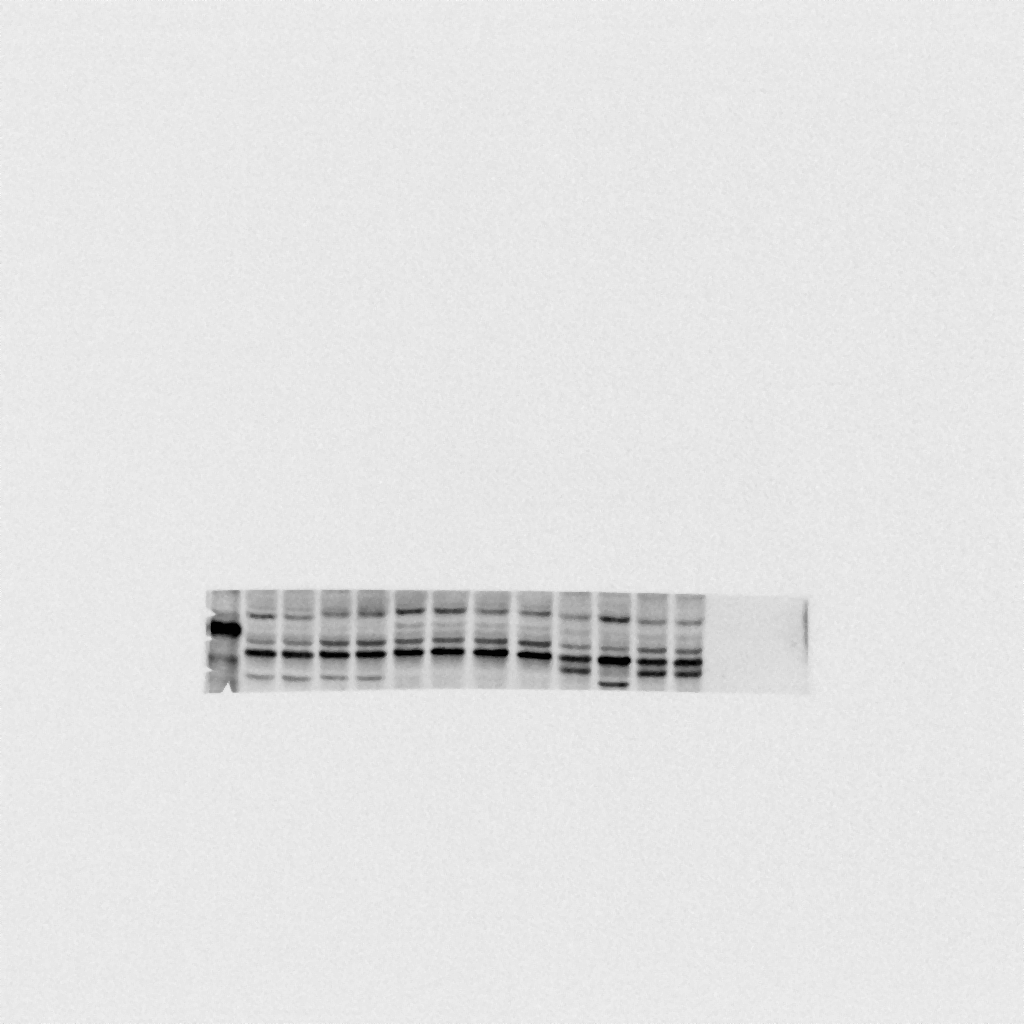


35 kDa -

25 kDa -

Spectra™ Multicolor Broad Range Protein Ladder Thermo Scientific™

UCP3 (33 kDa)

Coq9^+/+^

6-7 MO

Coq9^+/+^

10-16 MO

Coq9^+/+^

>22 MO

Coq9^Q95X^

6-7 MO

Coq9^Q95X^

10-16 MO

Coq9^Q95X^

>22 MO


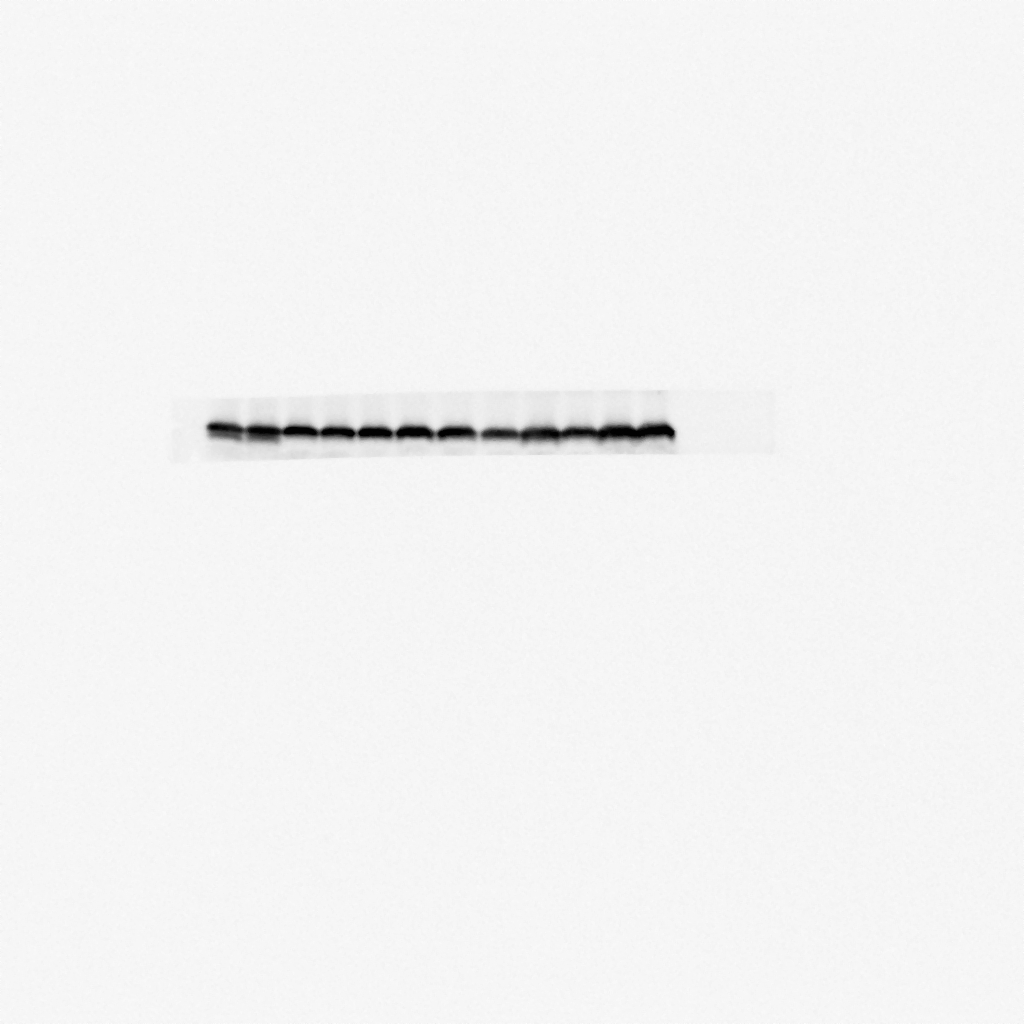


15 kDa -

Tom20 (16 kDa)

Note: lines 2, 3, 4, 5, 6, 7, 8, 9, 10, 11, 12, 13 in Figure 4E in the main text.

Figure S3. Leves of FGF21.

Coq9^Q95X^

>22 MO

Coq9^Q95X^

10-16 MO

Coq9^Q95X^

6-7 MO

Coq9^+/+^

>22 MO

Coq9^+/+^

10-16 MO

Coq9^+/+^

6-7 MO

FGF21 (GAPDH)

FGF21 precursor (25 kDa)

FGF21 mature (22 kDa)


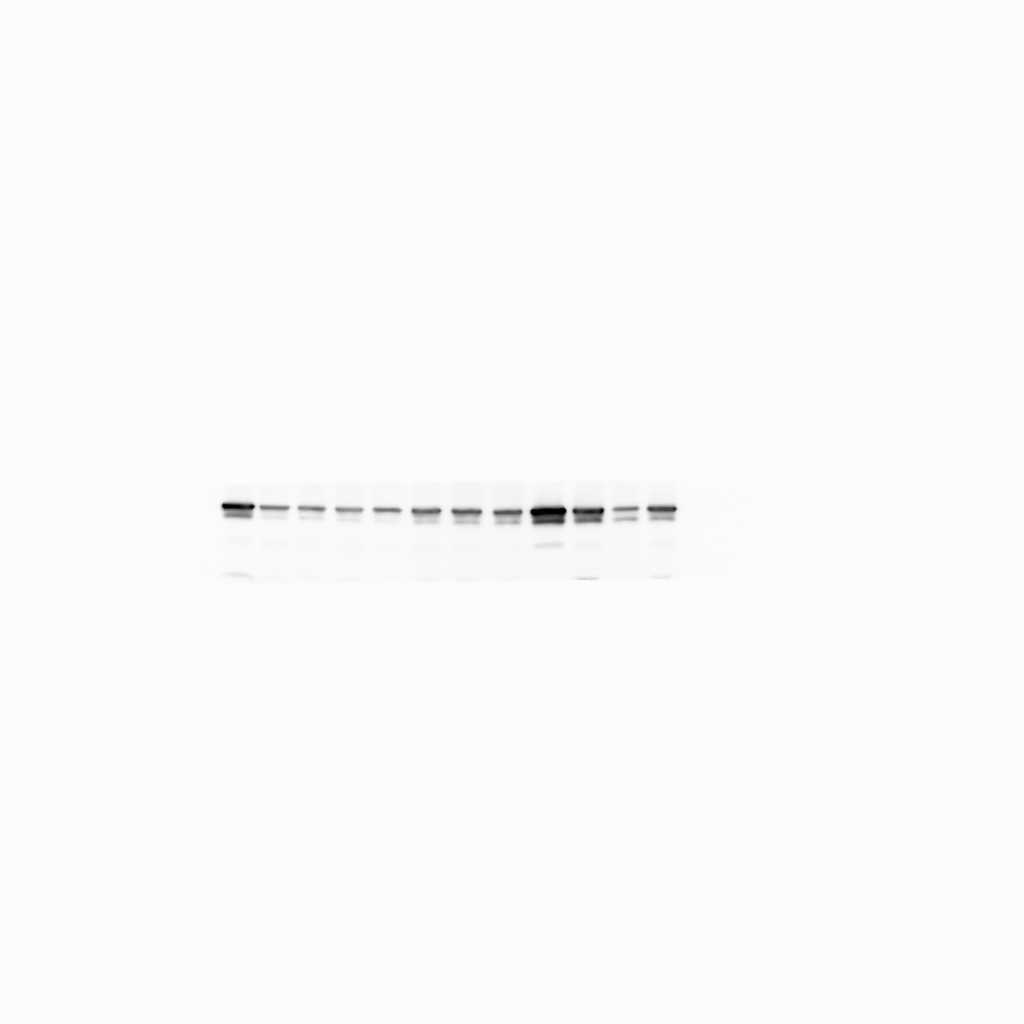


25 kDa -

Spectra™ Multicolor Broad Range Protein Ladder Thermo Scientific™

Coq9^Q95X^

>22 MO

Coq9^Q95X^

10-16 MO

Coq9^Q95X^

6-7 MO

Coq9^+/+^

>22 MO

Coq9^+/+^

10-16 MO

Coq9^+/+^

6-7 MO


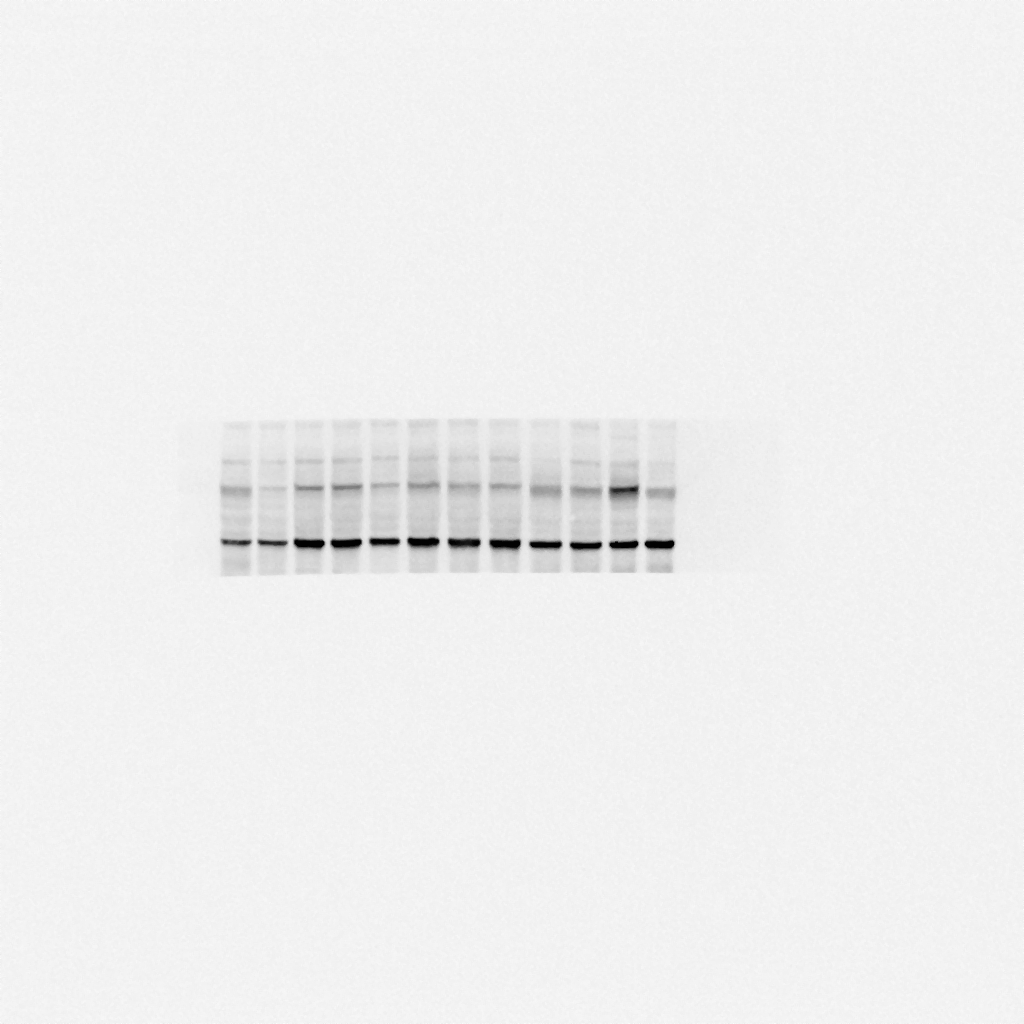


35 kDa -

GAPDH (37 kDa)

Note: lines 2, 3, 4, 5, 6, 7, 8, 9, 10, 11, 12, 13 in Figure S1 in the main text.

Figure S4A. Levels of proteins involved in the mitochondrial stress responses.

Coq9^Q95X^

>22 MO

Coq9^Q95X^

10-16 MO

Coq9^Q95X^

6-7 MO

Coq9^+/+^

>22 MO

Coq9^+/+^

10-16 MO

Coq9^+/+^

6-7 MO


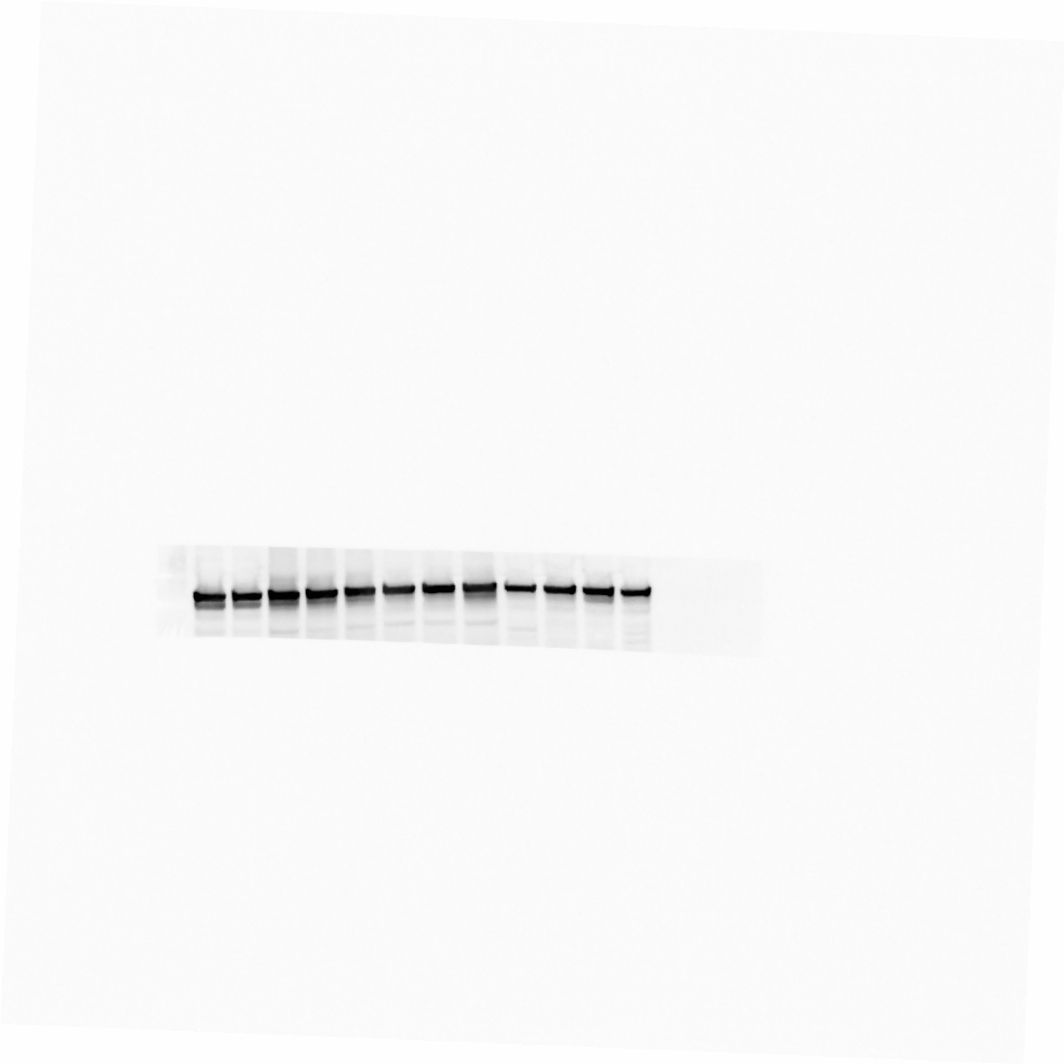


70 kDa -

Spectra™ Multicolor Broad Range Protein Ladder Thermo Scientific™

GRP75 (75 kDa)

Tom20 (16 kDa)

Coq9^Q95X^

>22 MO

Coq9^Q95X^

10-16 MO

Coq9^Q95X^

6-7 MO

Coq9^+/+^

>22 MO

Coq9^+/+^

10-16 MO

Coq9^+/+^

6-7 MO


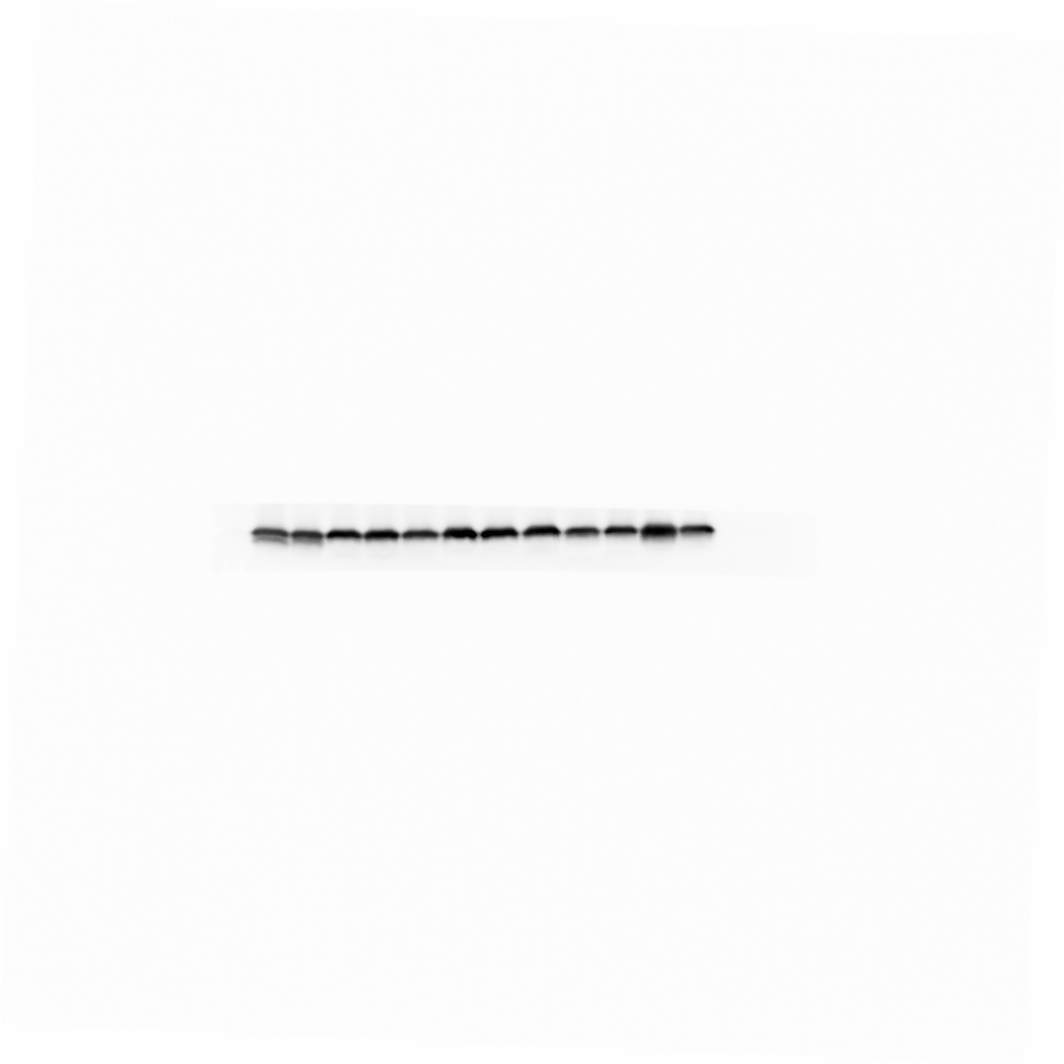


15 kDa -

Note: lines 2, 3, 4, 5, 6, 7, 8, 9, 10, 11, 12, 13 in Figure S2A in the main text.

Figure S4B. Levels of proteins involved in the mitochondrial stress responses.

Coq9^Q95X^

>22 MO

Coq9^Q95X^

10-16 MO

Coq9^Q95X^

6-7 MO

Coq9^+/+^

>22 MO

Coq9^+/+^

10-16 MO

Coq9^+/+^

6-7 MO

CLPP


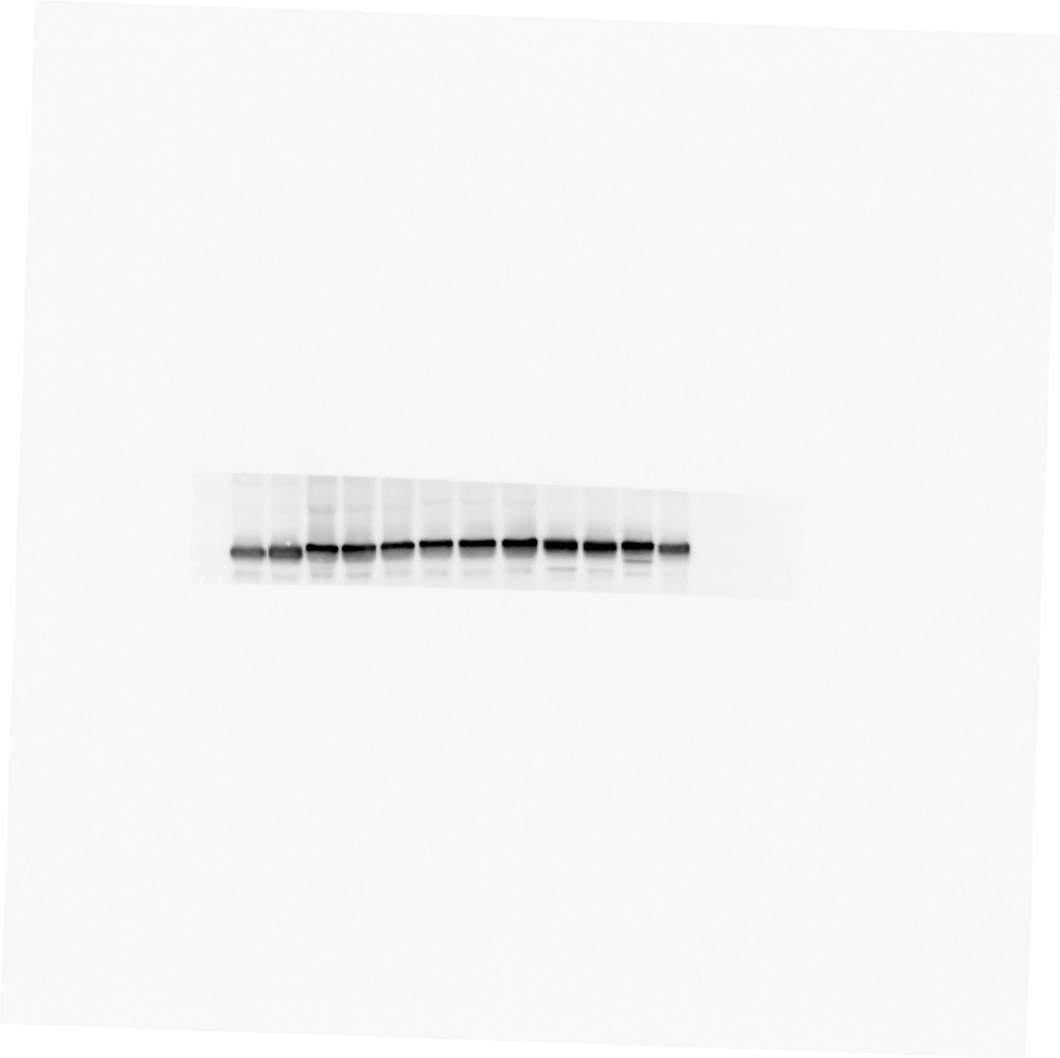


35 kDa -

25 kDa -

Spectra™ Multicolor Broad Range Protein Ladder Thermo Scientific™

CLPP (30 kDa)

Coq9^Q95X^

>22 MO

Coq9^Q95X^

10-16 MO

Coq9^Q95X^

6-7 MO

Coq9^+/+^

>22 MO

Coq9^+/+^

10-16 MO

Coq9^+/+^

6-7 MO


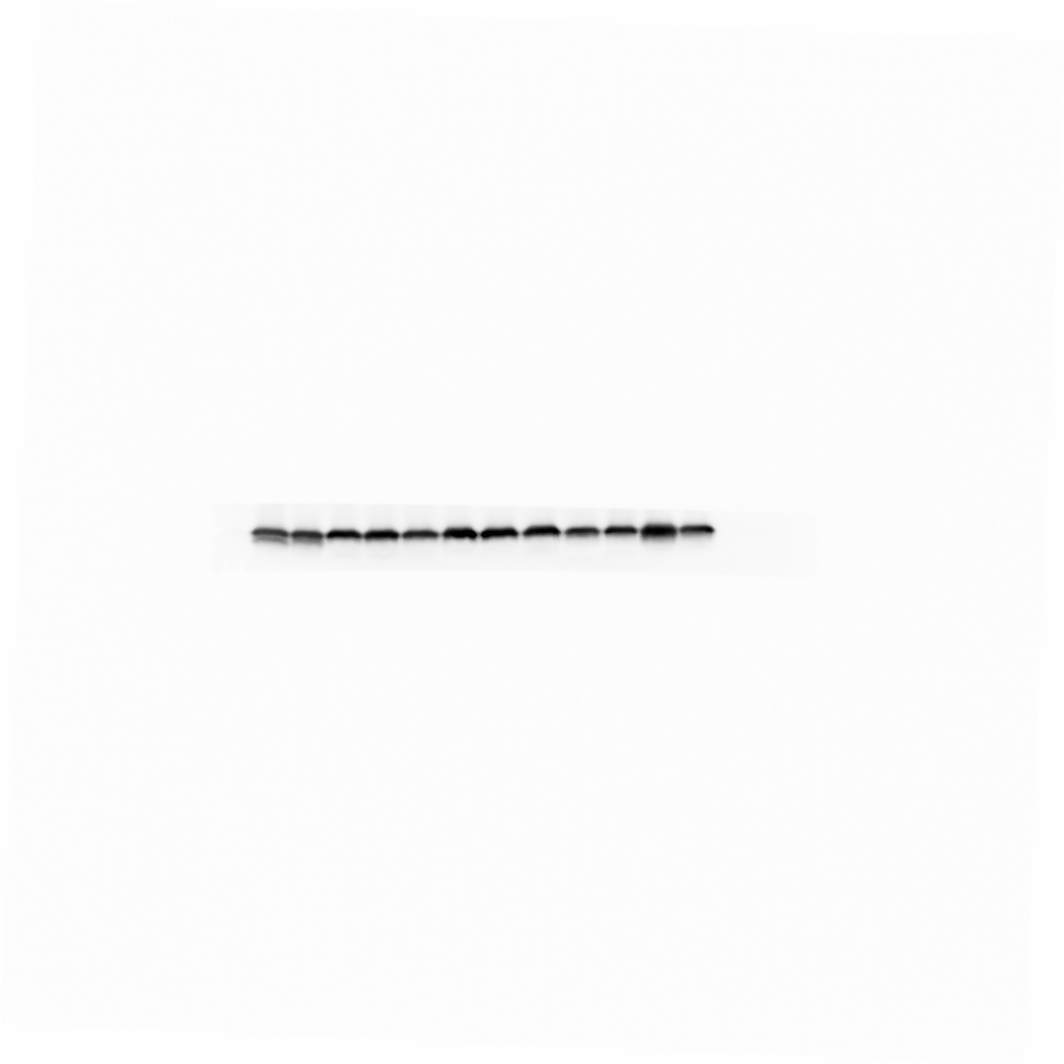


15 kDa -

Tom20 (16 kDa)

Note: lines 2, 3, 4, 5, 6, 7, 8, 9, 10, 11, 12, 13 in Figure S2B in the main text.
